# Phylogenetic relationships and biogeography of Asia *Callicarpa* (Lamiaceae), with consideration of a long-distance dispersal across the Pacific Ocean —insights into divergence modes of pantropical flora

**DOI:** 10.3389/fpls.2023.1133157

**Published:** 2023-05-15

**Authors:** Huimin Cai, Xing Liu, Wenqiao Wang, Zhonghui Ma, Bo Li, Gemma L. C. Bramley, Dianxiang Zhang

**Affiliations:** ^1^Department of Agricultural College, State Key Laboratory for Conservation and Utilization of Subtropical Agro-bioresources, Guangxi Key Laboratory of Sugarcane Biology, Guangxi University, Nanning, Guangxi, China; ^2^College of Agronomy, Jiangxi Agricultural University, Nanchang, Jiangxi, China; ^3^Herbarium, Royal Botanic Gardens, Kew, London, United Kingdom; ^4^South China Botanical Garden, Chinese Academy of Sciences, Guangzhou, Guangdong, China

**Keywords:** *Callicarpa*, phylogeny, historical biogeography, amphi-Pacific tropical disjunction, long-distance dispersal

## Abstract

There are about 140 species of *Callicarpa* L. 1753 (Lamiaceae), with more species richness in tropical to subtropical Asia and the New World. The genus might provide an insight into the amphi-Pacific disjunction pattern of tropical and subtropical vegetation. This study has greatly improved the phylogenetic underpinning for *Callicarpa*, derived from more inclusive taxonomic samplings, and employing data on both two-nuclear and eight-chloroplast regions. To address time and patterns of diversification in *Callicarpa*, we conducted divergence time and biogeographic analyses, and inferred shifts in the distribution areas across the phylogenetic clades. Our phylogenetic results show that *Callicarpa* is monophyletic with respect to the groups considered, and eight well-supported primary clades were discerned in the combined analyses. Our estimates indicated that the crown group of *Callicarpa* originates around the Late-Eocene (ca. 36.23 Ma) and diversification within most clades is concentrated in the Miocene and continued to the Pleistocene. In addition, our biogeographic analyses suggested that the probable ancestor of the Callicarpa crown clade originated in East Asia and Southeast Asia. Multiple dispersal and vicariance events contributed to the current distribution of the taxa. Furthermore, this genus expanded eastward out of East and Southeast Asia to the New World by long-distance dispersal, which inspired us to better understand the amphi-Pacific disjunct distribution.

## 1 Introduction

Understanding how distributions of organisms have been shaped is a fundamental question in biogeography and the use of molecular clocks and fossil records introduced a timeframe for the evolution of the taxa (Smith and Peterson, 2002). Complex interactions between abiotic and biotic factors and geological-tectonic settings have played an important role in this process. Amphi-Pacific disjunctions is a striking distribution pattern in biogeography, and temperate elements between eastern Asia and North America have been extensively studied in plants (Wen, 2001; Donoghue and Smith, 2004; Wen et al., 2016). These have intrigued many botanists and biogeographers to produce more studies of Northern Hemisphere botanical biogeography (Gaynor et al., 2020; Zhang et al., 2020b; Zhou et al., 2020). Wen (2001) suggested that the disjunction pattern was largely caused by complex processes such as dispersal, extinction, speciation, vicariance, and stasis. The relevant disjunction patterns are common in plants, and many temperate forest groups originated and diversified within East Asia, followed by movements out of Asia to the New World (Wen, 1999; Manos and Donoghue, 2001; Milne and Abbott, 2002; Donoghue and Smith, 2004). Two major hypotheses have been proposed to explain amphi-Pacific tropical disjunctions —the boreotropics hypothesis and the West Gondwanan vicariance hypothesis (Yang et al., 2017). The boreotropics hypothesis postulates a continuous belt of tropical to subtropical forest at middle to northern latitudes of the Northern Hemisphere, and the continents were connected by the Bering and North Atlantic land bridges during the early Cenozoic (Wolfe, 1975; Tiffney, 1985; Lavin and Luckow, 1993; Wen et al., 2016). Recently, an increased focus on the amphi-Pacific tropical (subtropical) disjunction of taxa has supplemented our knowledge of how this distribution pattern has been achieved. *Dendropanax* (Araliaceae) is disjunctly distributed in tropical to subtropical Asia and the Neotropics, and Li and Wen (2013) hypothesized that the genus originated in the Old World and migrated to the New World *via* the North Atlantic land bridges in the early Tertiary. Yang et al. (2018) suggested a Eurasian origin of Sabiaceae (with an amphi-Pacific tropical disjunct distribution) in the late Cretaceous, and a boreotropical range expansion during the Paleogene and a long distance dispersal from Central America to tropical Asia during the Neogene and Quaternary boundary in *Kingsboroughia alba*. Lian et al. (2020) inferred that the formation and breakup of the boreotropical floral may have been responsible for the amphi-Pacific disjunct distribution within Pachygoneae. The West Gondwanan vicariance hypothesis postulates a tropical origin and expansion in southern West Gondwana followed by vicariance from tectonic separation into South America and Africa (Yang et al., 2017), and has not been applied to any taxa showing the amphi-Pacific tropical distribution pattern. Similar distribution patterns were also reported within groups such as Leydigiopsis species (Van Damme and Sinev, 2013), Symplocaceae (Fritsch et al., 2015), *Diplazium* (Wei et al., 2015), and diving beetles (Toussaint et al., 2017). Also, disjunct species ranges could be explained by long-distance dispersal and it is known that a great variety of processes can move seeds by anemochory, hydrochory, autochory, ectozoochory, and endozoochory (Van Der Pijl, 1982; Higgins et al., 2003).

**Figure 1:**
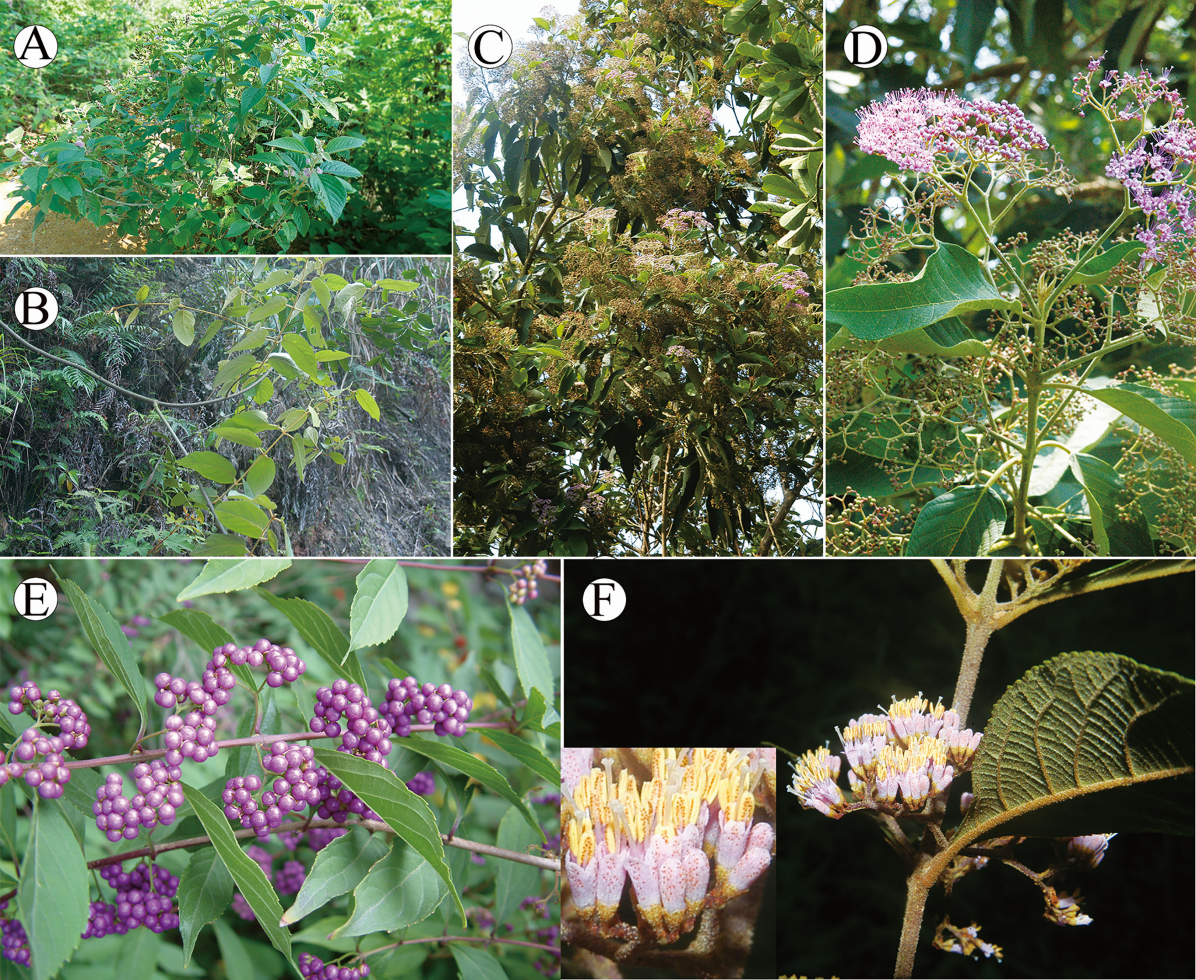
Morphology of *Callicarpa*. **(A)** erect shrub showed by *C. candicans*; **(B)** woody climber showed by *C. integerrima* var. *chinensis*; **(C)** tree showed by *C. nudiflora*; **(D)** Cymose or thyrsoid inflorescence, axillary showed by *C. nudiflora*; **(E)** purple fruits showed by *C. dichotoma*; **(F)** red glands on the inflorescences showed by *C. bodinieri*.

*Callicarpa* (Lamiaceae), with the nickname ‘beauty berry’, was first described by Linnaeus (1753). The genus takes its name because of its attractive purple fruits usually displaying in the autumn (**Figure 1**). There are about 140 species of *Callicarpa* in temperate, subtropical, and tropical Asia, America, Australia, and the Pacific Islands (Leeratiwong et al., 2007; Bramley, 2009; Bramley, 2013; Ma et al., 2016). However, regional diversity is variable and the genus is more species rich in the Old World, particularly with *ca.* 51 species in Malaysia (Bramley, 2013) and *ca.* 48 species in China (Chen and Gilbert, 1994). The current distribution range of *Callicarpa* recognized species over the world is characterized by a typical East Asia-Southeast Asia/North America disjunction, especially in tropical and subtropical regions on both sides of the Pacific Ocean. Under this biogeographic pattern, differentiation history of *Callicarpa* might be a good candidates for biogeographic studies in pantropical plants. A tropical and subtropical amphi-Pacific disjunction is among the most fascinating distribution patterns, and what might have been responsible for this pattern is the focus of most research into this disjunction. East and Southeast Asia are pivotal, having two of the highest levels of species diversity in the Northern Hemisphere owing to their geological and climatological history (Latham and Ricklefs, 1993; Wu and Wu, 1996; Myers et al., 2000; Tan et al., 2020; Zhang et al., 2020b). During their evolutionary history, *Callicarpa* may have exhibited significant species diversification in Southeast Asia and East Asia. Equally importantly, most species of this genus are much-valued traditional medicinal plants, and research has tended to focus on several of them at present, such as *C. bodinieri*, *C. macrophylla*, *C. kwangtungensis*, *C. nudiflora*, and *C*. *integerrima* (Wu et al., 2018; Ma et al., 2022). Based on current research results, *Callicarpa* produce abundant flavonoid, terpenoid, and phenylethanol glycosides, and have significant pharmacological effects on the prevention and treatment of health disorders such as inflammation, menoxenia, hematuria, and scrofula (Tu et al., 2013; Yang et al., 2021).

To date, no comprehensive molecular phylogenetic study on the infrageneric system of the genus has been presented. Here, we display the first comprehensive molecular investigation of the genus *Callicarpa* in Asia and discuss the infrageneric phylogenetic relationships. Our samples were mainly collected from China and Southeast Asia, alongside individual species expanding to Korea, New Guinea, Australia, and America. Currently improved phylogenetic underpinning is warranted to identify the factors responsible for shaping the present distribution of *Callicarpa* and gain new insights into the patterns of diversification of this genus in the World. The reconstruction of ancestral areas on a phylogeny is important for understanding the biogeographical history of a lineage, as it permits the inference of the place of origin and dispersal routes of organisms. In a sense, a better understanding of biogeographic history within *Callicarpa* could also provide a case for exploring biogeographic patterns in amphi-Pacific tropical disjunctions.

In this study, our objectives are (1) to reconstruct phylogenetic relationships within *Callicarpa* using eight chloroplast and two nuclear DNA regions, thus with a more comprehensive sampling than in previous studies; (2) reveal the timing of genus and species differentiation; (3) investigate the historical biogeography of the *Callicarpa*, a pantropical flora.

## 2 Materials and methods

### 2.1 Taxon sampling

A total of 145 accessions representing 56 recognized species and several unidentified species ingroups were collected in this study. Among them were four varieties represented by *C. bodinieri* var. *rosthornii*, *C. integerrima* var. *chinensis*, *C. pedunculata* var. *longifolia* and *C. longifolia* var. *lanceolaria*, and four forms represented by *C. rubella* f. *angustata*, *C. rubella* f. *crenata*, *C. japonica* f. *kiiruninsularis*, and *C. brevipes* f. *annamensis*. We selected *Dasymalla teckiana*, *Dicrastylis parvifolia*, *Clerodendrum* sp., *Clerodendrum cyrtophyllum*, *Gomphostemma Chinense*, and *Vitex negundo* as the outgroups. To estimate molecular divergence times of the *Callicarpa* group, we expanded our sampling more broadly across Lamiaceae to ensure sufficient representation for assigning appropriate fossil calibrations. Our sampling also encompassed the mainly biogeographic range of *Callicarpa*, including representatives from Asia, Australia, New Guinea, and America. The original sources of the plant materials used in this study and voucher information are presented in additional files (**Table 1**). We added fifteen sequences representing five species from Ocimeae, eight species from Mentheae, and two from Elsholtzieae, and the DNA sequence of closely related species were used to substitute for the several species lacking corresponding data (Li et al., 2016). All the data presented in the study are deposited in the NCBI database (https://www.ncbi.nlm.nih.gov/) (**Table 2**).

**Table 1:** Taxa sampled and the vouchers in infrageneric phylogenetic analysis of *Callicarpa.*

Taxon	Voucher	Locality	DNA No.
*C. americana*	K-LCD_081-84-00-507 (K)	#	14293
*C. americana*	ZHMa 0101 (IBSC)	Xishuangbanna, Yunnan, China	MZH76
*C. poilanei*	Suddee et al. 2596 (BKF)	Ubon Ratchathani, Nam Yuen dist, Thailand	23191
*C. angustifolia*	Leeratiwong 05-195 (KKU)	Trat, Thailand	38160
*C. angustifolia*	H. Toyama et al. 2221 (Kyushu University)	Kampot, Cambodia	2221
*C. angustifolia*	H. Toyama et al. 1431 (Kyushu University)	Kampot, Cambodia	1431
*C. angustifolia*	H. Toyama et al. 774 (Kyushu University)	Kho Khong, Cambodia	774
*C. maingayi*	Leeratiwong 05-193 (KKU)	Narathiwat, Thailand	38169
*C. furfuracea*	S. Tagane et al. MY364 (Kyushu University)	Tanintharyi, Myanmar	MY364
*C. furfuracea*	Leeratiwong 06-325 (KKU)	Songkhla, Thailand	38163
*C. furfuracea*	S. Tagane et al. T4789 (Kyushu University)	Nakhon Si Thammarat, Thailand	T4789
*C. arborea*	Nguyen et al. HNK720 (K)	Hoa Binh Dist, Vietnam	23189
*C. arborea*	Leeratiwong 05-261 (KKU)	Mae Hong Son, Thailand	38161
*C. arborea*	ZHMa 071 (IBSC)	Luxi, Yunnan, China	MZH5
*C. arborea*	ZHMa 092 (IBSC)	Napo, Guangxi, China	MZH41
*C. arborea*	ZHMa 099 (IBSC)	Xishuangbanna, Yunnan, China	MZH42
*C. arborea*	ZHMa 096 (IBSC)	Tianbao, Yunnan, China	MZH75
*C. arborea*	LB 0240 (IBSC)	Yunnan, China	MZH29
*C. arborea*	S. Tagane et al. T1412 (Kyushu University)	Chiang Mai, Thailand	T1412
*C.* sp1	Leeratiwong 05-232 (KKU)	Loei, Thailand	38173
*C.* sp2	de Kok 1275 (K, LAE)	from Bulolo to Lea, Papua New G	38174
*C. yunnanensis*	ZHMa 0114 (IBSC)	Xishuangbanna, Yunnan, China	MZH77
*C. erioclona*	Nguyen et al. HNK934 (K)	Nam Cat Tien NP, Vietnam	23190
*C. erioclona*	J. Gagul 9 (K)	Papua, Indonesia	42085
*C. candicans*	Leeratiwong 04-119 (KKU)	Songkhla, Thailand	38162
*C. candicans*	W.W Nong VTN828 (IBSC)	Vietnam	MZH61
*C. candicans*	ZHM 0128 (IBSC)	Bawanglin, Hainan, China	MZH62
*C. candicans*	LB 0162 (IBSC)	Hainan, China	MZH20
*C.* sp3	S. Tagane et al. T3048 (Kyushu University)	Pechaburi, Thailand	T3048
*C.* sp5	S. Tagane et al. V2677 (Kyushu University)	Thua Thien Hue, Vietnam	V2677
*C. luteopunctata*	ZHMa 0162 (IBSC)	Emei, Sichuan, China	MZH52
*C. luteopunctata*	G. Yao 402 (IBSC)	Emei, Sichuan, China	MZH53
*C. integerrima* var. *chinensis*	X.X. Huang 8887 (IBSC)	Longnan, Jiangxi, China	MZH19
*C. integerrima* var. *chinensis*	X.X. Huang 1032 (IBSC)	Rucheng, Hunan, China	MZH60
*C. integerrima* var. *chinensis*	X.X. Huang 1016 (IBSC)	Rucheng, Hunan, China	MZH59
*C. dichotoma*	ZHM 0126 (IBSC)	Wugong mountain, Jiangxi, China	MZH38
*C. dichotoma*	ZHM 080 (IBSC)	SCBG, China	MZH2
*C. peichieniana*	X. Guo 101 (IBSC)	Huizhou, Guangdong, China	MZH81
*C. macrophylla*	Leeratiwong 05-262 (KKU)	Chiang Mai, Thailand	38168
*C. macrophylla*	ZHMa 087 (IBSC)	Kunming, Yunnan, China	MZH18
*C. macrophylla*	ZHMa 0158 (IBSC)	Shanglin, Guangxi, China	MZH56
*C. macrophylla*	ZHMa 095 (IBSC)	Funing, Yunnan, China	MZH57
*C. macrophylla*	17010 (IBSC)	Zhaoqin, Guangdong, China	MZH58
*C. nudiflora*	ZHMa 0155 (IBSC)	Changjiang, Hainan, China	MZH69
*C. nudiflora*	ZHMa 0112 (IBSC)	Xishuangbanna, Yunnan, China	MZH70
*C. nudiflora*	N. Nguyen et al. V3120 (Kyushu University)	Da Nang, Vietnam	V3120
*C. nudiflora*	L.X. Zhou 5208 (IBSC)	Lingshui, Hainan, China	MZH71
*C. kochiana*	G. Yao 255 (IBSC)	Shaoguan, Guangdong, China	MZH10
*C. kochiana*	LB (IBSC)	Dongguan, Guangdong, China	MZH49
*C. kochiana*	X.X. Huang 8697 (IBSC)	Yujiang, Jiangxi, China	MZH50
*C. kochiana*	X.X. Huang 1025 (IBSC)	Rucheng, Hunan, China	MZH51
*C. loboapiculata*	ZHMa 013 (IBSC)	Sanfang, Guangxi, China	MZH14
*C. loboapiculata*	ZHMa 0143 (IBSC)	SCBG, China	MZH34
*C. longifolia*	Leeratiwong 05-249 (KKU)	Songkhla, Thailand	38165
*C. longifolia*	de Kok 1029 (K)	Along Sungei Imbak, Malaysia	21551
*C. longifolia*	R.J. Johns 9851 (K)	Papua, Indonesia	42087
*C. longifolia*	P.W. Xie 10-145 (IBSC)	Xishuangbanna, Yunnan, China	MZH28
*C. longifolia*	LB 0242 (IBSC)	Yunnan, China	MZH31
*C. longifolia*	S. Tagane et al. 5830 (Kyushu University)	Kampot, Cambodia	5830
*C. longifolia*	T. Yahara et al. V3294 (Kyushu University)	Ha Tinh, Vietnam	V3294
*C. longifolia*	ZHMa 0117 (IBSC)	Xishuangbanna, Yunnan, China	MZH43
*C. longifolia*	T. Yahara & D. Darnaedi S413 (Kyushu University)	Bantimulung Bulusarum, Indonesia	S413
*C. longifolia*	T. Yahara et al. IK133 (Kyushu University)	Mandor, Indonesia	IK133
*C. longifolia*	H. Toyama et al. 2329 (Kyushu University)	Kampot, Cambodia	2329
*C. longifolia*	S. Tagane et al. V1723 (Kyushu University)	Khanh Hoa, Vietnam	V1723
*C. longifolia*	Nob. Tanaka et al. MY105 (Kyushu University)	Shan, Myanmar	MY105
*C. longifolia*	H. Toyama et al. 2227 (Kyushu University)	Kampot, Cambodia	2227
*C. longifolia*	C.J. Yang et al. V2321 (Kyushu University)	Khanh Hoa, Vietnam	V2321
*C. longifolia* var. *lanceolaria*	Leeratiwong 04-014 (KKU)	Loei, Thailand	38166
*C. longifolia* var. *lanceolaria*	ZHM 0154 (IBSC)	Ningming, Guangxi, China	MZH65
*C. longissima*	ZHMa 0159 (IBSC)	Changjiang, Hainan, China	MZH72
*C. pedunculata*	J. Halford GAQLD0430 (K)	QLD, Conondale, Australia	42088
*C. angusta*	Leeratiwong 06-291 (KKU)	Quang Ninh, Vietnam	38159
*C.* sp4	S. Tagane et al. IK1596 (Kyushu University)	Bukit Bangkirai, Indonesia	IK1596
*C. albida*	T. Yahara et al. IJ266 (Kyushu University)	West Java, Indonesia	IJ266
*C. acutidens*	T. Yahara et al. V5569 (Kyushu University)	Ha Tinh, Vietnam	V5569
*C. bodinieri*	Zhuqiu Song ZHM0125 (IBSC)	Honghe, Yunnan, China	MZH40
*C. bodinieri*	ZHM 094 (IBSC)	Napo, Guangxi, China	MZH39
*C. bodinieri*	ZHMa 053 (IBSC)	Longnan, Jiangxi, China	MZH3
*C. bodinieri*	ZHMa 0423 (IBSC)	Lingchuan, Guangxi, China	MZH27
*C. bodinieri*	#	#	22401
*C. bodinieri* var. *rosthornii*	X.X. Huang 8418 (IBSC)	Longnan,Jiangxi, China	MZH66
*C. brevipes*	ZHMa 0130 (IBSC)	Dongguan, Guangdong, China	MZH44
*C. brevipes*	ZHMa 0331 (IBSC)	Ledong, Hainan, China	MZH46
*C. brevipes*	S. Tagane et al. V3825 (Kyushu University)	Ha Tinh, Vietnam	V3825
*C. brevipes* f. *annamensis*	T. Yahara et al. V2379 (Kyushu University)	Thua Thien Hue, Vietnam	V2379
*C. brevipes* f. *annamensis*	T. Yahara et al. V2723 (Kyushu University)	Thua Thien Hue, Vietnam	V2723
*C. formosana*	ZHMa 083 (IBSC)	SCBG, China	MZH1
*C. formosana*	G. Yao 261 (IBSC)	Shaoguan,Guangdong, China	MZH33
*C. formosana*	ZHMa 059 (IBSC)	Longnan, Jiangxi, China	MZH36
*C. formosana*	ZHMa 0104 (IBSC)	Xishuangbanna, Yunnan, China	MZH37
*C. formosana* var. *longifolia*	ZHMa 0107 (IBSC)	Xishuangbanna, Yunnan, China	MZH74
*C. giraldii*	LB 0233 (IBSC)	Yunnan, China	MZH12
*C. giraldii*	ZHMa 072 (IBSC)	Longlin, Yunnan, China	MZH21
*C. giraldii*	ZHMa 0122 (IBSC)	Kunming, Yunnan, China	MZH64
*C. glandulosa*	Leeratiwong 04-105 (KKU)	Loei, Thailand	38164
*C. hainanensis*	ZHMa 079 (IBSC)	SCBG, China	MZH22
*C. japonica*	K-LCD_1934-12904	#	14294
*C. japonica*	ZHMa 0160 (IBSC)	Korea	MZH35
*C. japonica*	#	#	25990
*C. japonica*	#	#	22402
*C. japonica* f. *kiruninsularis*	ZHM 002 (IBSC)	SCBG, China	MZH15
*C. kwangtungensis*	ZHMa 0124 (IBSC)	Xiangtan, Hunan, China	MZH73
*C. mollis*	# (K)	#	22403
*C. mollis*	S. Tagane et al. T4475 (Kyushu University)	Loei, Thailand	T4475
*C. pauciflora*	ZHMa 090 (IBSC)	Shaoguan,Guangdong, China	MZH80
*C. rubella*	S. Tagane et al. T4590 (Kyushu University)	Loei, Thailand	T4590
*C. rubella*	H. Toyama et al. V1867 (Kyushu University)	Lam Dong, Vietnam	V1867
*C. rubella*	T. Yahara et al. V5740 (Kyushu University)	Ha Tinh, Vietnam	V5740
*C. rubella*	Leeratiwong 05-252 (KKU)	Phetchabun, Thailand	38171
*C. rubella*	Nguyen et al. HNK106 (K)	Sa Pa, Vietnam	23188
*C. rubella*	ZHMa 089 (IBSC)	Shenzhen, China	MZH6
*C. rubella*	G. Yao 258 (IBSC)	Shaoguan,Guangdong, China	MZH32
*C. rubella* f. *angustata*	ZHMa 064 (IBSC)	Dawei mountain,Yunnan, China	MZH11
*C. rubella* f. *angustata*	W.W Nong VTN670 (IBSC)	Vietnam	MZH25
*C. rubella* f. *angustata*	LB 0241 (IBSC)	Yunnan, China	MZH30
*C. rubella* f. *angustata*	S. Tagane et al. V4086 (Kyushu University)	Lam Dong, Vietnam	V4086
*C. rubella* f. *crenata*	W.W Nong VTN654 (IBSC)	Vietnam	MZH24
*C. rubella* f. *crenata*	W.W Nong VTN654 (IBSC)	Vietnam	MZH23
*C. rubella* f. *crenata*	ZHM 014 (IBSC)	Sanfang, Guangxi, China	MZH83
*C. cathayana*	X.X.Huang 8942 (IBSC)	Jiulian mountain,Jiangxi, China	MZH17
*C. cathayana*	X.X.Huang 8918 (IBSC)	Jinpanshan, Jiangxi, China	MZH54
*C. prolifera*	C.M.Tan 91152 (IBSC)	Wuyi mountain, Jiangxi, China	MZH82
*C. erythrosticta*	X.X.Huang 8700 (IBSC)	Jinpanshan, Jiangxi, China	MZH68
*C. longipes*	ZHMa 085 (IBSC)	Huizhou, Guangdong, China	MZH9
*C. longipes*	B. Li 0069 (IBSC)	Dongguan, Guangdong, China	MZH47
*C. longipes*	X.X. Huang 8919 (IBSC)	Yujiang, Jiangxi, China	MZH48
*C. stapfii*	Bramley et al. SAN147250 (K, SAN)	Silau Silau trail, Kinabalu NP, Malaysia	25529
*C. stapfii*	S. Tagane et al. SWK1216 (Kyushu University)	Sarawak, Malaysia	SWK1216
*C. havilandii*	S. Tagane & U. Shimizu-kaya SWK2627 (Kyushu University)	Miri, Malaysia	SWK2627
*C. havilandii*	T. Yahara et al. SWK473 (Kyushu University)	Sarawak, Malaysia	SWK473
*C. havilandii*	T. Yahara et al. SWK451 (Kyushu University)	Sarawak, Malaysia	SWK451
*C. havilandii*	Bramley et al. SAN1472 (K, SAN)	Bukit Silam, Malaysia	25524
*C. hispida*	Bramley et al. SAN1472 (K, SAN)	Danum, Malaysia	25525
*C. pentandra*	E. Suzuki et al. IK895 (Kyushu University)	Serimbu, Indonesia	IK895
*C. pentandra*	S. Tagane & U. Shimizu-kaya SWK2639 (Kyushu University)	Miri, Malaysia	SWK2639
*C. pentandra*	T. Yahara et al. SWK1024 (Kyushu University)	Sarawak, Malaysia	SWK1024
*C. pentandra*	Bramley et al. SAN1472 (K, SAN)	Bombalai Hill, Malaysia	25527
*C. pentandra*	Leeratiwong 06-333 (KKU)	Narathiwat, Thailand	38170
*C. pentandra*	C. Barker 131 (K)	Papua, Indonesia	42089
*C. pentandra*	T.M.A. Utteridge 701 (K)	Papua, Indonesia	42092
*C. pentandra*	Bramley GB60 (K)	Sumatra, Bolian, Indonesia	42093
*C. pentandra*	#	#	25526
*C. scandens*	Bramley et al. SAN1472 (K, SAN)	Danum Valley Field Centre, Malaysia	25528
*C. acuminata*	1936 (K)	Belize, Central America	1936
Outgroups			
*Clerodendrum* sp.	J. Liu LJ501	#	LJ501
*Clerodendrum cyrtophyllum*	J. Liu LJ9	#	LJ9
*Gomphostemma chinense*	J. Liu LJ510	#	LJ510
*Vitex negundo*	J. Liu LJ268	#	LJ268
*Dasymalla teckiana*	#	Australian National Botanic Gardens (ANBG), Australia	#
*Dicrastylis parvifolia*	#	Australian National Botanic Gardens (ANBG), Australia	#

**Table 2:** GeneBank accession numbers for *Callicarpa* and representation assigning fossil calibration.

Taxa	ITS accession	ETS	matk	psbJ_petA	rpL32_trnL	trnD_T	trnG_trnS	trnH_psbA	trnQ_rps16	trnV-ndhC
Callicarpa										
*C. americana* 14293	ON820115	ON931484	OP032108	#	#	OP734891	OP735028	OP735071	OP734977	OP744560
*C. americana* MZH76	OM333866	OM307559	OM630187	#	#	OM403780	OM403863	#	OM403947	OM307487
*C. angusta* 38159	OM333840	OM307533	OM630158	#	OM501607	OM403748	OM403831	OM473325	OM403915	OM307473
*C. poilanei* 23191	#	OM307603	OM530154	OM439786	OM501603	OM403792	OM403827	OM439784	OM403911	OM307462
*C. angustifolia* 38160	OM333834	OM307525	OM630159	OM460787	OM501608	OM403749	OM403832	#	OM403916	OM307467
*C. arborea* 23189	ON820116	ON931485	OP032109	OP081532	#	OP734892	OP735030	OP735074	OP734981	OP744564
*C. arborea* 38161	ON820117	ON931486	OP032111	OP081533	OP734844	OP734893	#	OP735046	OP734947	OP744536
*C. arborea* MZH5	ON820118	ON931487	OP032110	OP081534	#	OP734894	#	OP735078	OP734983	OP744568
*C. arborea* MZH41	OM333841	OM307534	#	OM460817	OM501611	OM403754	OM403837	OM489764	OM403921	OM307474
*C. arborea* MZH42	ON820119	ON931488	OP032112	OP032161	OP081584	OP734895	OP032171	#	OP734952	OP744540
*C. arborea* MZH75	ON820120	ON931489	OP032113	OP081535	OP734845	OP734896	OP735027	OP735070	OP734976	OP744559
*C. arborea* MZH29	#	#	#	OP081582	OP734890	OP734897	#	OP735077	#	OP744567
*C. bodinieri* MZH3	ON820121	#	OP032114	OP081536	OP734846	OP734898	OP735005	OP735052	OP734953	OP744541
*C. bodinieri* var. *rosthornii* MZH66	OM333842	OM307535	OM630167	OM460816	OM501612	OM403755	OM403838	OM489769	OM403922	OM403728
*C. brevipes* MZH27	ON820122	ON931490	#	OP081537	OP734847	OP734899	#	OP735079	#	#
*C. brevipes* MZH44	ON820123	ON931491	OP032115	#	OP081586	OP734900	#	OP032167	#	#
*C. candicans* 38162	ON820124	ON931492	OP032116	OP081538	OP734848	OP734901	OP735001	OP735047	OP734948	OP744537
*C. candicans* MZH61	ON820125	ON931493	OP032117	OP081539	OP734849	OP734902	OP735007	#	OP734955	OP744543
*C. candicans* MZH62	ON820126	ON931494	OP032118	OP081540	OP734850	OP734903	OP735008	#	OP734956	OP744544
*C. candicans* MZH20	OM333844	OM307537	OM630169	OM460814	OM501614	OM403757	OM403840	OM473348	OM403924	OM307476
*C. erioclona* 23190	OM333830	OM307521	OM530155	OM439785	OM439782	OM403744	OM403826	OM439783	OM403910	OM307461
*C. erioclona* 42085	ON820127	ON931495	#	#	#	OP734904	OP735035	OP735086	OP734989	OP744572
*C. formosana* MZH1	OM333848	OM307541	#	OM460811	OM501618	OM403761	OM403844	OM473337	OM403928	OM307479
*C. formosana* MZH33	ON820128	ON931496	OP032119	OP081541	OP734851	#	OP735011	OP735054	OP734959	OP744547
*C. formosana* MZH36	#	ON931497	#	OP081542	#	OP734905	#	OP735080	OP734984	OP744569
*C. formosana* MZH37	ON820129	ON931498	OP032120	OP081543	#	OP734906	OP735033	OP735081	#	#
*C. formosana* var. *longifolia* MZH74	OM333869	OM307562	OM630190	OM460790	#	OM403783	OM403866	OM489774	OM403950	OM403730
*C. furfuracea* 38163	OM638741	OM307526	OM630217	OM460819	OM501609	OM403750	OM403833	OM473326	OM403917	OM307468
*C. giraldii* MZH12	ON820130	ON931499	OP032121	OP032160	OP081585	OP734907	OP032170	OP032165	OP734966	OP744551
*C. giraldii* var. *giraldii* MZH21	OM333868	OM307561	OM630189	OM460789	OM530213	OM403782	OM403865	OM473349	OM403949	OM307489
*C. giraldii* var. *subcanescens* MZH64	#	ON931500	OP032122	OP081544	OP734852	OP734908	OP735034	OP735082	OP734985	#
*C. glandulosa* 38164	#	OM307604	OM630218	OM460796	OM530211	OM403778	OM403861	OM473327	OM403946	#
*C. hainanensis* MZH22	OM333849	OM307542	OM630173	OM460810	OM501619	OM403762	OM403845	OM473350	OM403929	OM403725
*C. hainanensis* MZH46	OM333843	OM307536	OM630168	OM460815	OM501613	OM403756	OP032173	OM489765	OM403923	OM307475
*C. hispida* 25524	OM333835	OM307527	OM530152	OM460821	OM501604	OM403745	OM403828	#	OM403912	OM403737
*C. hispida* 25525	OM333836	OM307528	OM530153	OM460820	OM501605	OM403746	OM403829	#	OM403913	OM403738
*C. integerrima* var. chinensis MZH19	OM333838	OM307530	OM630183	OM460802	OM501631	OM403773	OM403857	OM473347	OM403941	OM307470
*C. integerrima* var. chinensis MZH60	ON820131	ON931501	OP032123	OP081545	OP734853	OP734909	OP735022	OP735066	OP734972	OP744557
*C. integerrima* var. *chinensis* MZH59	ON820132	ON931502	OP032124	#	OP734854	OP734910	#	OP735084	OP734988	#
*C. dichotoma* MZH38	ON820133	ON931503	OP032125	OP081546	OP734855	OP734911	OP735010	OP735053	OP734958	OP744546
*C. dichotoma* MZH2	OM333846	OM307539	OM630171	OM460812	OM501616	OM403759	OM403842	OM473338	OM403926	OM307478
*C. japonica* 25990	#	ON931504	#	OP081547	OP734856	#	OP735032	OP735076	OP734982	OP744566
*C. japonica* 22402	#	ON931505	#	OP081548	OP081587	#	OP735029	OP735073	OP734980	OP744563
*C. japonica* f. *kiruninsularis* MZH15	OM333850	OM307543	OM630174	OM460809	OM501620	OM403763	OM403846	OM473345	OM403930	OM403724
*C. kochiana* MZH10	OM333852	OM307545	#	OM473322	#	OM403820	OM403905	OM473341	OM403987	OM307481
*C. kochiana* MZH49	ON820134	ON931506	OP032126	OP081549	OP734857	OP734912	OP735012	OP735055	OP734960	OP744548
*C. kochiana* MZH50	ON820135	ON931507	OP032156	OP081550	OP734858	OP734913	OP735013	OP735056	OP734961	OP744549
*C. kochiana* MZH51	#	OM307551	OP032127	#	OP734859	OP734914	#	OP735083	OP734986	OP744570
*C. kwangtungensis* MZH73	OM333853	OM307546	OM630175	#	OM501622	OM403764	OM403848	OM489773	OM403932	OM307482
*C. loboapiculata* MZH14	OM333854	OM307547	OM630176	OM460807	OM501623	OM403765	OM403849	OM473344	OM403933	OM403723
*C. loboapiculata* MZH34	ON820136	ON931508	OP032128	OP081551	OP734860	#	OP735014	OP735057	OP734962	#
*C. longifolia* 38165	ON820137	ON931509	OP032129	OP081552	OP734861	OP734915	OP735002	OP735048	#	OP744538
*C. longifolia* 21551	#	ON931510	OP032130	OP081553	OP734862	OP734916	OP734999	OP735044	OP734944	#
*C. longifolia* 42087	#	ON931511	#	#	#	OP734917	OP735036	OP735087	OP734990	OP744573
*C. longifolia* MZH28	ON820138	ON931512	#	#	OP734863	OP734918	#	OP735058	#	#
*C. longifolia* MZH31	ON820139	ON931513	#	OP081554	OP734864	#	OP735015	OP735059	OP734963	OP744550
*C. longifolia* MZH43	#	ON931514	OP032131	OP032162	#	OP734919	OP032172	OP032166	OP734987	OP744571
*C. longifolia* var. *lanceolaria* 38166	ON820140	ON931515	OP032132	#	OP734865	OP734920	OP735003	OP735049	OP734949	#
*C. longipes* MZH9	OM333856	OM307549	OM630178	OM460805	OM501625	OM403767	OM403851	OM473340	OM403935	OM403721
*C. longipes* MZH47	ON820145	ON931516	#	OP081555	OP081583	OP734921	#	OP735060	OP734964	#
*C. longipes* MZH48	ON820144	ON931517	OP032133	OP081556	OP734866	OP734922	OP735016	#	OP734965	#
*C. longissima* MZH72	OM333857	OM307550	OM630423	#	OM501626	OM403768	OM403852	OM489772	OM403936	OM307484
*C. luteopunctata* MZH52	ON820143	ON931518	OP032134	OP081557	OP734867	OP734923	OP735017	#	OP734967	#
*C. luteopunctata* MZH53	OM333858	OM307551	OM630179	OM460804	OM501627	OM403769	OM403853	OM489766	OM403937	OM403727
*C. macrophylla* 38168	ON820142	ON931519	OP032135	OP081558	OP734868	OP734924	OP735004	OP735050	OP734950	OP744539
*C. macrophylla* MZH18	ON820141	ON931520	OP032136	OP081559	OP734869	OP734925	OP735018	OP735061	OP734968	OP744552
*C. macrophylla* MZH56	OM333859	OM307552	OM630180	OM460803	OM501628	OM403770	OM403854	OM489767	OM403938	OM307485
*C. macrophylla* MZH57	ON820146	ON931521	OP032137	OP081560	OP734870	OP734926	OP735019	OP735062	OP734969	OP744553
*C. macrophylla* MZH58	ON820147	ON93152	OP032138	OP081561	OP734871	OP734927	OP735021	OP735065	OP734971	OP744556
*C. macrophylla* MZH71	ON820148	ON931523	OP032139	OP081562	OP734872	OP734928	OP735020	OP735064	#	OP744555
*C. maingayi* 38169	OM333833	OM307524	OM630164	OM460786	#	OM403779	OM403835	OM473328	#	OM307466
*C. mollis* 22403	#	OM307532	#	OM460798	#	#	#	OM460785	OM403945	OM307472
*C. bodinieri* 22401	#	ON931524	#	OP081563	OP734873	#	#	OP735072	OP734979	OP744562
*C. nudiflora* MZH69	OM333860	OM307553	OM630181	OM460801	OM501629	OM403771	OM403855	OM489771	OM403939	OM307486
*C. nudiflora* MZH70	ON820149	ON931525	OP032140	OP081564	OP734874	OP734929	#	OP735063	OP734970	OP744554
*C. pauciflora* MZH80	#	#	#	#	#	OM403822	#	OM489776	#	OM403732
*C. pedunculata* 42088	OM333839	OM307531	#	OM460791	#	OM403785	OM403867	OM473331	OM403951	OM307471
*C. peichieniana* MZH81	OM333861	OM307554	OM630182	#	OM501630	OM403772	OM403856	OM489777	OM403940	#
*C. pentandra* 25527	ON820150	ON931526	OP032141	OP032158	OP081588	OP734930	OP032169	OP032164	OP734946	OP744535
*C. pentandra* 38170	OM333837	OM307529	OM630165	OM460818	OM501610	OM403751	OM403829	OM473329	OM403918	OM307469
*C. pentandra* 42089	ON820151	ON931527	#	OP081565	OP734875	OP734931	OP735037	#	OP734991	OP744574
*C. pentandra* 42092	ON820152	ON931528	#	OP081566	OP734876	OP734932	OP735038	OP735088	OP734992	OP744575
*C. pentandra* 42093	ON820153	ON931529	OP032142	OP081567	OP734877	OP734933	#	OP735089	OP734993	#
*C. rubella* 38171	ON820154	ON931530	OP032143	OP081568	OP734878	#	#	OP735051	OP734951	#
*C. rubella* 23188	ON820155	ON931531	OP032155	OP081569	OP734879	OP734934	OP735000	OP735045	OP734945	#
*C. rubella* MZH6	OM333864	OM307557	ON964474	OP032159	OM530212	OM403821	OM403906	OM473339	OM403988	#
*C. rubella* MZH32	ON820156	ON931532	OP032144	OP081570	OP734880	OP734935	OP735026	#	OP734975	OP744558
*C. rubella* f. *angustata* MZH11	OM333862	OM307555	#	OM460800	OM501632	OM403774	OM403858	OM473342	OM403942	OM403722
*C. rubella* f. *angustata* MZH25	ON820157	ON931533	OP032145	OP081571	OP734881	OP734936	OP735023	OP735067	OP734973	#
*C. rubella* f. *angustata* MZH30	ON820158	ON931534	OP032146	OP081572	OP734882	#	OP735024	OP735068	#	#
*C. rubella* f. *crenata* MZH24	ON820159	ON931535	OP032147	OP081573	OP734883	#	OP735025	OP735069	OP734974	#
*C. rubella* f. *crenata* MZH35	OM333851	OM307544	#	OM460808	OM501621	#	OM403847	OM489762	OM403931	OM307480
*C. rubella* var. *subglabra* MZH23	OM333863	OM307556	OM630184	OM460799	OM501633	OM403775	OM403859	OM489761	OM403943	OM403726
*C. scandens* 25528	OM333828	OM307520	#	#	OM501606	OM403747	OM403830	OM473324	OM403914	OM307463
*C.* sp1 38173	OM333832	OM307523	OM630166	#	OM530176	OM403752	OM403835	#	OM403919	OM307465
*C.* sp2 38174	OM333831	OM307522	OM630426	OM460822	OM530177	OM403753	OM403836	OM473330	OM403920	OM307464
*C. yunnanensis* MZH77	OM333865	OM307558	OM630185	#	OM501634	OM403776	OM403860	OM489775	OM403944	OM403731
*C. bodinieri* MZH40	ON820160	ON931536	OP032148	OP081574	OP734884	OP734937	OP735006	#	OP734954	OP744542
*C. bodinieri* MZH39	OM333867	OM307560	OM630188	OM460788	#	OM403781	OM403864	OM489763	OM403948	OM307488
*C. prolifera* MZH82	OM333870	OM307564	#	#	#	#	#	OM489778	#	#
*C. rubella* f. *crenata* MZH83	#	ON931537	#	OP081575	#	OP734938	#	OP735085	#	#
*C. cathayana* MZH54	ON820161	ON931538	OP032149	OP081576	OP734885	OP734939	OP735009	#	OP734957	OP744545
*C. erythrosticta* MZH68	OM333847	OM307540	OM630172	#	OM501617	OM403760	OM403843	OM489770	OM403927	OM403729
*C. cathayana* MZH17	OM333845	OM307538	OM630170	OM460813	OM501615	OM403758	OM403841	OM473346	OM403925	OM307477
*C. japonica* 14294	ON820162	ON931539	OP032150	#	#	#	OP032168	OP032163	OP734978	OP744561
*C. stapfii* 25529	OM333829	#	OM630186	OM460797	#	OM403777	#	#	#	#
*C. pentandra* 25526	#	ON931540	#	OP081577	#	#	OP735031	OP735075	#	OP744565
*C. longifolia* var. *lanceolaria* MZH65	#	OM307563	#	#	#	OM403784	#	OM489768	#	#
*C. brevipes f. annamensis* V2379	#	#	#	#	OM530190	OM403824	OM403908	OM489792	OM403990	OM403733
*C. longifolia* V2321	OM333881	OM307590	OM630162	#	OM530189	OM403786	OM403868	#	OM403952	OM307510
*C. rubella* V1867	#	OM307601	OM630216	OM489760	OM530188	OM403787	OM403869	OM489791	OM403953	OM307519
*C. rubella* V5740	#	OM307581	OM630191	OM489755	OM530201	OM403788	OM403870	OM489804	OM403954	OM307502
*C. arborea* T1412	OM333871	OM307569	OM630192	#	OM530202	OM403789	OM403871	OM489786	#	OM307492
*C. furfuracea* MY364	#	#	OM630193	OM489757	OM530181	OM403790	OM403872	#	OM403955	OM307498
*C. rubella* f. *angustata* V4086	#	OM307602	#	OM489756	OM530198	OM403791	OM403873	OM489801	OM403956	OM403735
*C. longifolia* 5830	OM333888	OM307599	OM630161	OM489754	OM530174	#	OM403874	OM460783	OM403957	OM307517
*C.* sp3 T3048	OM333872	OM307571	OM630422	OM460792	#	OM403793	OM403875	OM489787	OM403958	OM307494
*C. nudiflora* V3120	#	OM307582	OM630194	OM489753	OM530194	OM403794	OM403876	OM489796	OM403959	OM307503
*C. longifolia* V3294	#	OM307589	OM630195	OM460793	OM530196	OM403795	OM403877	OM489798	OM403960	OM307509
*C. brevipes* V3825	OM333877	OM307583	OM630196	OM460794	OM530197	OM403796	OM403878	OM489799	OM403961	OM307504
*C. mollis* T4475	#	OM307580	#	OM460795	OM530185	#	OM403879	OM585503	OM403962	#
*C. anguifolia* 2221	#	OM307574	OM630197	OM460751	OM530170	#	OM403881	OM460779	OM403964	OM307497
*C. anguifolia* 1431	OM333874	OM307573	OM630160	#	#	OM403798	OM403882	OM460778	OM403965	OM307496
*C. acutidens* V5569	OM333878	OM307584	OM630163	#	OM530200	OM403799	OM403883	OM489803	OM403966	OM403736
*C. furfuracea* T4789	#	OM307575	OM630198	OM489750	OM530187	OM403800	OM403884	OM489789	OM403967	OM307499
*C. longifolia* 2227	#	OM307587	OM630200	OM489748	OM530171	OM403802	OM403886	OM460780	OM403969	OM307505
*C. longifolia* MY105	#	OM307598	OM630202	OM489746	OM530180	OM403804	OM403888	OM473336	OM403971	OM307516
*C. albida* IJ266	#	OM403739	#	OM473323	#	OM403823	OM403907	OM473332	#	OM403720
*C. longifolia* 2329	OM333883	OM307592	OM630203	OM489745	OM530172	OM403805	OM403889	OM460781	OM403972	OM307512
*C. brevipes* f. *annamensis* V2723	#	#	#	#	OM530193	OM403825	OM403909	OM489795	OM403991	OM403734
*C. rubella* T4590	#	OM307579	#	OM489743	OM530186	OM403807	OM403891	OM489788	OM403974	OM307501
*C. longifolia* S413	OM333882	OM307591	OM630204	OM489742	OM530182	OM403808	OM403892	OM489779	OM403975	OM307511
*C. angustifolia* 774	OM333873	OM307572	OM630205	OM489741	OM530204	OM403809	OM403893	OM460777	OM403976	OM307495
*C.* sp4 IK1596	OM333886	OM307596	OM630207	OM489739	OM530205	OM403811	OM403895	OM473335	#	OM307515
*C. pentandra* IK895	#	OM307577	OM630208	#	OM530179	OM403812	OM403896	OM473334	OM403978	#
*C. longifolia* IK133	OM333884	OM307594	OM630209	OM489738	OM530178	OM403813	OM403897	OM473333	OM403979	OM307513
*C. pentandra* SWK2639	OM333876	OM307578	OM630210	OM489737	OM530206	OM403814	OM403898	OM489785	OM403980	#
*C. havilandii* SWK2627	#	OM307567	OM630211	OM489736	OM530207	OM403815	OP735043	OP735094	OP734998	#
*C. stapfii* SWK1216	#	OM307565	#	OM489735	OM530208	#	OM403900	OM489783	#	#
*C. pentandra* SWK1024	OM333875	OM307576	#	OM489734	OM530184	OM403816	OM403901	OM489782	OM403982	OM307500
*C. havilandii* SWK473	#	OM307568	OM630212	OM489733	OM530209	OM403817	OM403902	OM489781	OM403983	OM307491
*C. havilandii* SWK451	#	OM307566	OM630213	OM473320	OM530183	OM403818	OM403903	OM489780	OM403984	OM307490
*C.* sp5. V2677	#	OM307570	OM630214	#	OM530192	OM403819	OM403904	OM489794	OM403985	OM307493
*C. longifolia* V1723	#	OM307593	OM630425	OM473321	#	#	#	OM489790	OM403986	#
Outgroup										
*Clerodendrum* sp.	ON820163	#	OP032152	OP081579	OP734887	OP734941	OP735040	OP735091	OP734995	OP744577
*Clerodendrum cyrtophyllum*	ON820164	#	OP032151	OP081578	OP734886	OP734940	OP735039	OP735090	OP734994	OP744576
*Gomphostemma Chinese*	ON820165	#	OP032153	OP081580	OP734888	OP734942	OP735041	OP735092	OP734996	OP744578
*Vitex negundo*	#	#	OP032154	OP081581	OP734889	OP734943	OP735042	OP735093	OP734997	OP744579
*Dasymalla teckiana*	#	#	NC_058334	NC_058334	NC_058334	NC_058334	NC_058334	NC_058334	NC_058334	NC_058334
*Dicrastylis parvifolia*	GQ381162	#	NC_058335	NC_058335	NC_058335	NC_058335	NC_058335	NC_058335	NC_058335	NC_058335
Elsholtzieae										
*Collinsonia canadensis*	JQ669087	JQ669157	KY624850	#	JQ669291	#	#	DQ667358	#	#
*Elsholtzia ciliata*	MH117518	JQ669170	KY624860	NC_050945	JQ669306	#	NC_050945	MH117072	NC_050945	NC_050945
Mentheae										
*Hyptis laniflora*	JF301548	JF304259	KJ772845	#	JQ669317	JF301606	MH612795	#	#	#
*Isodon dawoensis*	KF855429	MG232701	JF954204	MW018469	#	KF855759	#	#	MW018469	#
*Lavandula angustifolia*	FJ593399	#	HE967430	NC_046835	JQ669323	KF855779	NC_046835	#	#	NC_046835
*Melissa officinalis*	JF301353	JF301325	KP172051	MT634148	JQ669335	#	MT634148	MK090069	MT634148	MT634148
*Mentha arvensis*	JQ669115	JQ669190	KP172052	NC_044082	JQ669336	#	NC_044082	MH753577	KC591690	#
*Monarda citriodora*	JQ669124	JQ669200	MG225355	#	JQ669346	#	#	AY943563	#	#
*Neoeplingia leucophylloides*	JF301354	JF301327	#	#	JQ669348	#	#	#	#	#
*Nepeta cataria*	JQ669126	JQ669202	KT176606	MT663220	JQ669349	#	#	MH753573	MT663220	MT663220
Ocimeae										
*Ocimum basilicum*	MT338842	#	KX096054	MN687904	JQ669350	KF855776	MN687904	JX262185	MN687904	MN687904
*Plectranthus cremnus*	JQ230965	#	MF694872	#	#	KF855755	MH612752	#	#	MH884564
*Prunella vulgaris*	JQ669130	JQ669206	KJ593074	NC_039654	JQ669358	#	NC_039654	MH117244	NC_039654	NC_039654
*Rosmarinus officinalis*	KJ584197	JF301329	KP172065	NC_027259	JQ669364	#	NC_027259	#	NC_027259	NC_027259
*Salvia glutinosa*	KJ584253	KF307496	KP852741	K344723	JQ669372	#	KP852867	KX247541	MK344723	MK344723

### 2.2 DNA extraction and sequencing

Two nuclear [internal transcribed spacer (ITS) and external transcribed spacer (ETS)] and eight chloroplast [(*matK*, *rpl32*-*trnL*, *trnD*-*trnT*, *trnH*-*psbA*, *psbJ*-*petA*, *trnQ-*5’*rps16*, 3’*trnV-ndhC*, and *trnS (GCU)*-*trnG* intergenic spacer] regions were used in this study. Extracting total genomic DNA of the samples with silica dried leaf tissue followed the 2 × CTAB method of Doyle and Doyle (1987), and the DNeasy Plant Mini Kit (Qiagen, Hilden, Germany) was used for herbarium materials following the manufacturer’s instructions. Primer pairs used in polymerase chain reaction (PCR) amplification of the ten regions are listed in **Table S1** (White et al., 1990; Sun et al., 1994; Demesure et al., 1995; Sang et al., 1997; Baldwin and Markos, 1998; Beardsley and Olmstead, 2002; Tate and Simpson, 2003; Shaw et al., 2005; Andersson, 2006; Shaw et al., 2007; Dong et al., 2012). PCR products were assessed by electrophoresis on 2% agarose gel. The samples were sequenced on BGI’s sequencing platform both strands of DNA with overlapping regions. The raw sequences were initially edited with Sequencher v5.4.5 (Gene Codes Corporation, Ann Arbor, MI, USA) and then aligned in MAFFT v7.450 (Katoh and Standley, 2013) with manual adjustment where necessary in MEGA v7.0 (Kumar et al., 2016) and BioEdit v7.250 (Hall, 1999).

### 2.3 Phylogenetic inference

Phylogenetic analyses of the data matrices —two nuclear ribosomal DNA, eight chloroplast DNA and combined ten locus data —were conducted respectively using maximum likelihood (ML) and Bayesian inference (BI) methods. The ML phylogeny was reconstructed in the program Phylosuite v1.2.2 (Zhang et al., 2020a) with the Iqtree (Nguyen et al., 2015). The bootstrap (BS) percentage for each branch was estimated by running 1,000 bootstrap replicates. The BI analysis was conducted with MrBayes (Ronquist et al., 2012) in Phylosuite v1.2.2 (Zhang et al., 2020a) and implemented in CIPRES (http://www.phylo.org/) (Miller et al., 2010). For BI analysis, ModelFinder (Kalyaanamoorthy et al., 2017) was used for the selection of the most appropriate evolutionary model (nucleotide substitution model) (Edge-linked) using BIC criterion. The run with 10,000,000 generations was conducted. Four Markov chains with two runs were implemented and sampled every 1,000 generations, and the first 25% of all trees were regarded as ‘burn-in’. The majority consensus of the remaining trees was generated to show posterior probability (PP) support for clades. Convergence was determined in Tracer v1.7.1 (Rambaut et al., 2018) and was considered to be attained when ESS > 200 or when the average standard deviation of the split frequencies was < 0.01. The best-fit partition model for Iqtree and Mrbayes analysis are listed in **Table S2**.

### 2.4 Molecular divergence time estimation

Lineage divergence time provides important information for understanding biogeographic history. As there are no known fossils for *Callicarpa*, molecular dating in this study relies on fossils from related clades in Lamiaceae. Although Lamiaceae are also not well represented in the fossil record, there are accepted fossils that can be used as calibration points in related studies within the family. We used the following two fossils as calibration points (Drew and Sytsma, 2012; Li et al., 2017). The first one was suggested conservatively to place at the crown of Nepetoideae. It was based on the hexacolpate and three-nucleate pollen fossil from Early Eocene sediments in India, which was identified as *Ocimum* (Kar, 1996). The second fossil calibration point was the fruit fossil of *Melissa* from the Early-Middle Oligocene (Martinez-Millan, 2010), which was assigned to constrain the most recent common ancestor (MRCA) of *Melissa* and *Neoeplingia*. We used a reduced dataset of 56 samples keeping one accession per species. Divergence times were estimated using BEAST2 (Bouckaert et al., 2014) in CIPRES (http://www.phylo.org/) with the nuclear and plastid concatenated matrix, and before that we got the appropriate XML file by Beauti (part of the BEAST package). A Yule tree prior and the relaxed exponential clock model were selected. The substitution model for each partition was determined within the Akaike information criterion (AIC; Akaike, 1974) as implemented in the program MrMtgui (Nylander, 2004) (**Table S3**). Two calibration points we all used a lognormal distribution model. Additionally, the first fossil had an offset at 49 million years ago (Ma), a mean of 2.6 Ma, and a standard deviation (SD) of 0.5 Ma while the latter had an offset at 28.4 Ma (mean: 2.6 Ma, SD: 0.5 Ma). Markov Chain Monte Carlo chains were run for 400,000,000 generations with sampling every 40000 generations. Convergence and the adequate effective sample size values (>200) for the BEAST analysis was evaluated in Tracer v1.7 (Rambaut et al., 2018). We ran each dataset four times respectively and combined the tree files in Logcombiner (part of the BEAST package). After burn-in of 20%, the maximum clade credibility (MCC) tree with median branch lengths and 95% highest posterior density (HPD) intervals on nodes was calculated using TreeAnnotator (part of the BEAST package). To get a simple temporal dynamics of diversification of *Callicarpa*, we generated standard lineage-through-time (LTT) plots for the maximum clade credibility (MCC) tree of sampled species in the R package ‘ape’ (Paradis et al., 2004). To convert stratigraphic ages into absolute ages, we used the geological timescale (Walker et al., 2018).

### 2.5 Ancestral area reconstruction

For biogeographic reconstruction, we assigned *Callicarpa* species to eight areas based on their distribution records acquired from the literature, our own fieldwork, and herbarium records: A) East Asia; B) South Asia; C) Southeast Asia; D) Oceania (including Australia, New Guinea, New Zealand; E) Temperate North America; F) Neotropics (Cuba, Columbia, Peru and Bolivia); G) Islands of India Ocean (Mascarene Islands and Reunion Island); H) Pacific Islands. The analysis was implemented by statistical dispersal extinction cladogenesis (S-DEC) in RASP (Yu et al., 2015; Yu et al., 2020). We used the maximum clade credibility (MCC) tree with fossils calibration produced in BEAST to estimate the ancestral geographic ranges of *Callicarpa*. The trees from BEAST were used as input trees, and other parameters were set to their default.

## 3 Results

### 3.1 Phylogenetic relationships between infrageneric species

Topologies derived from cpDNA (eight regions) and nrDNA (two loci) were broadly congruent (**Figures S1-1, S1-2**), but better resolution and stronger branch support was achieved by combining the datasets. The phylogenetic trees using BI and ML analyses had nearly the same topology, only differing in the location of *C. peichieniana* (**Figures S2-1, S2-2**).

**Figure 2:**
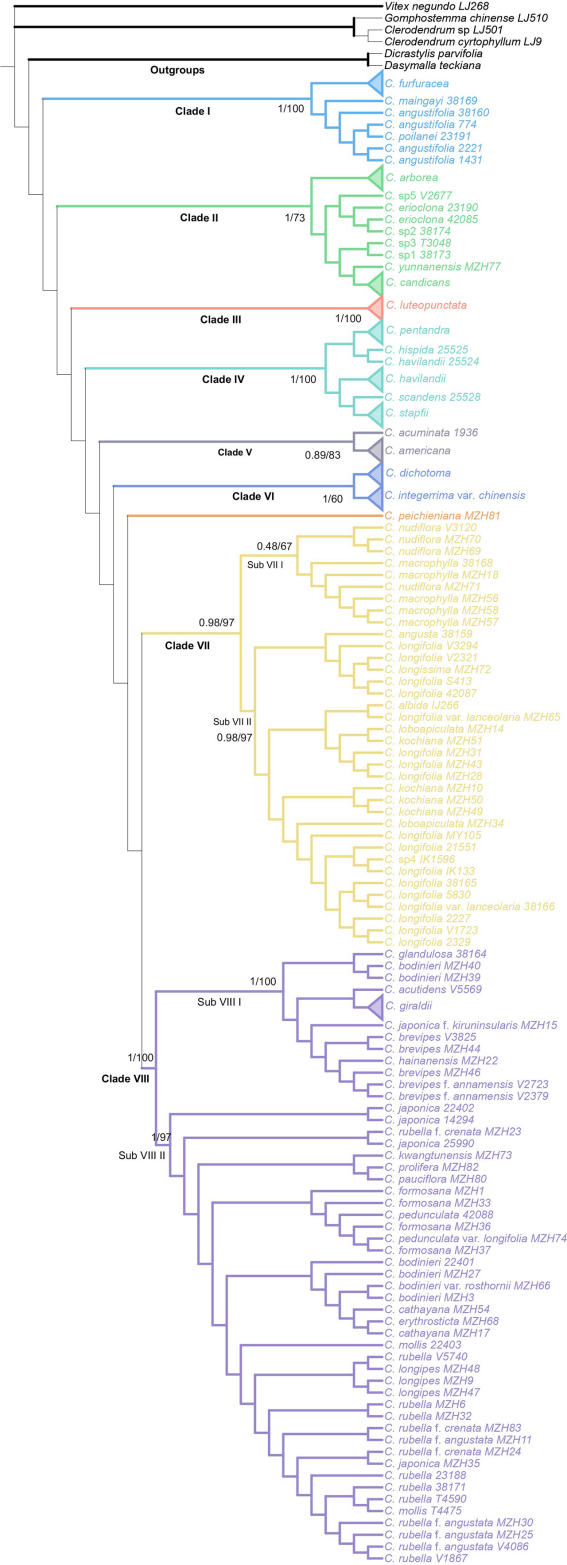
Bayesian consensus tree of *Callicarpa* based on the combined DNA data (two nuclear and eight chloroplast regions). Maximum likelihood bootstraps and Bayesian posterior probabilities of major clades are shown.

The combined analysis indicated that the genus *Callicarpa* was monophyletic (Posterior probability, PP=1.00; Bootstrap percentage, BP=100) with respect to the groups considered, and eight well-supported primary clades of the genus were resolved (**Figure 2**). Clade I (PP=1.00, BP=100) branched off first in the genus and was composed of some tropical species native to Southeast Asia with large anthers and short filaments. Clade II was comprised of tropical species, small trees species of *Callicarpa* (PP=1.00, BP=73). *Callicarpa luteopunctata* split from the remaining species and formed a separate branch (Clade III, PP=1.00, BP=100). This species has an extremely narrow geographical distribution and is only found in high altitude mountains of the Yunnan-Guizhou Plateau located in Southwest China. In addition, the species with variable flower parts and initially being of the genus *Geunsia* Blume formed a strongly supported group Clade IV (PP=1.00, BP=100). Two species from America formed an independent clade V. In BI analyses (**Figure S2-1**), Clade VI consisted of *C. dichotoma*, *C. integerrima* var. *chinensis* with good support (PP=0.99, BP=97), which represented the rare lianas or slender-climbing species of this genus, and *C. peichieniana* forming a single branch (PP=0.53), while in the ML tree (**Figure S2-2**), three species comprised Clade VI (BP=60). It’s a pity that their positions weren’t resolved well. Clade VII was also a well (moderately) supported clade (PP=0.98, BP=97) consisting of two subclades. Subclade VII_I_ consisted of two subtropic species with robust cymes (*C. macrophylla* and *C. nudiflora*). Subclade VII_II_ was characterized by a conspicuous interpetiolar ridge and white baccate drupe, and the tree indicated the paraphyletic status of *C. longifolia*. Clade VIII was recognized as one broad clade, which was strongly supported as the largest group of *Callicarpa* investigated in the present study (PP=1.00, BP=100) and comprised some taxa with a broad range of variation in morphological characters. This clade mostly represented small shrubs with simple slender cyme and when more than one accession of a species was applied, they were either exclusive lineages or grouped together with more closely related species in a particular clade or more than one small group (e.g., *C. brevipes*, *C. giraldii*, *C. bodinieri*, *C. formosana* and *C. rubella*).

### 3.2 Divergence time estimation and biogeographical reconstruction

The divergence time estimates based on nrDNA+cpDNA datasets were here reported and used for further biogeographical analysis. The BEAST analysis indicated that the crown group age of *Callicarpa* was estimated at 36.23 Ma (95% HPD: 18.81–60.39 Ma) around the Late-Eocene and diversification within most clades concentrated in the Miocene and continued to the Pleistocene (**Figure 3**, node 1). Our dating suggested that *C. americana* was the first to split from the remaining species in the Middle-Oligocene, *ca.* 27.9 Ma (**Figure 3**, node 2). The crown age of the tropical occurring, small trees species of *Callicarpa* was inferred to be *ca.* 17.03 Ma (95% HPD: 7.35–28.3 Ma) (**Figure 3**, node 3). There was an early middle Miocene crown age for several native species in Southeast Asia, *ca.* 16.51 Ma (95% HPD: 3.42–33.78 Ma) and *ca.* 16.14 Ma (95% HPD: 6.88–26.98 Ma), respectively (**Figure 3**, node 4, node 5). The ages of the crown nodes of the two great groups were estimated to be at a similar time, *ca.* 16.51 Ma (95% HPD: 9.48-25.16 Ma) (**Figure 3**, node 6) and *ca.* 13.53 Ma (95% HPD: 6.46-22.37 Ma) (**Figure 3**, node 7). Furthermore, the divergence time of major species are listed in **Table S4**. The LTT plot for *Callicarpa* overall is shown in **Figure 3-1**. Before about the middle-Miocene (*ca.* 14 Ma), there was a relatively stable diversification rate and then a subsequently rapid diversification (accelerated lineage accumulation). This upward trend was maintained during the Pliocene and the Pleistocene.

**Figure 3:**
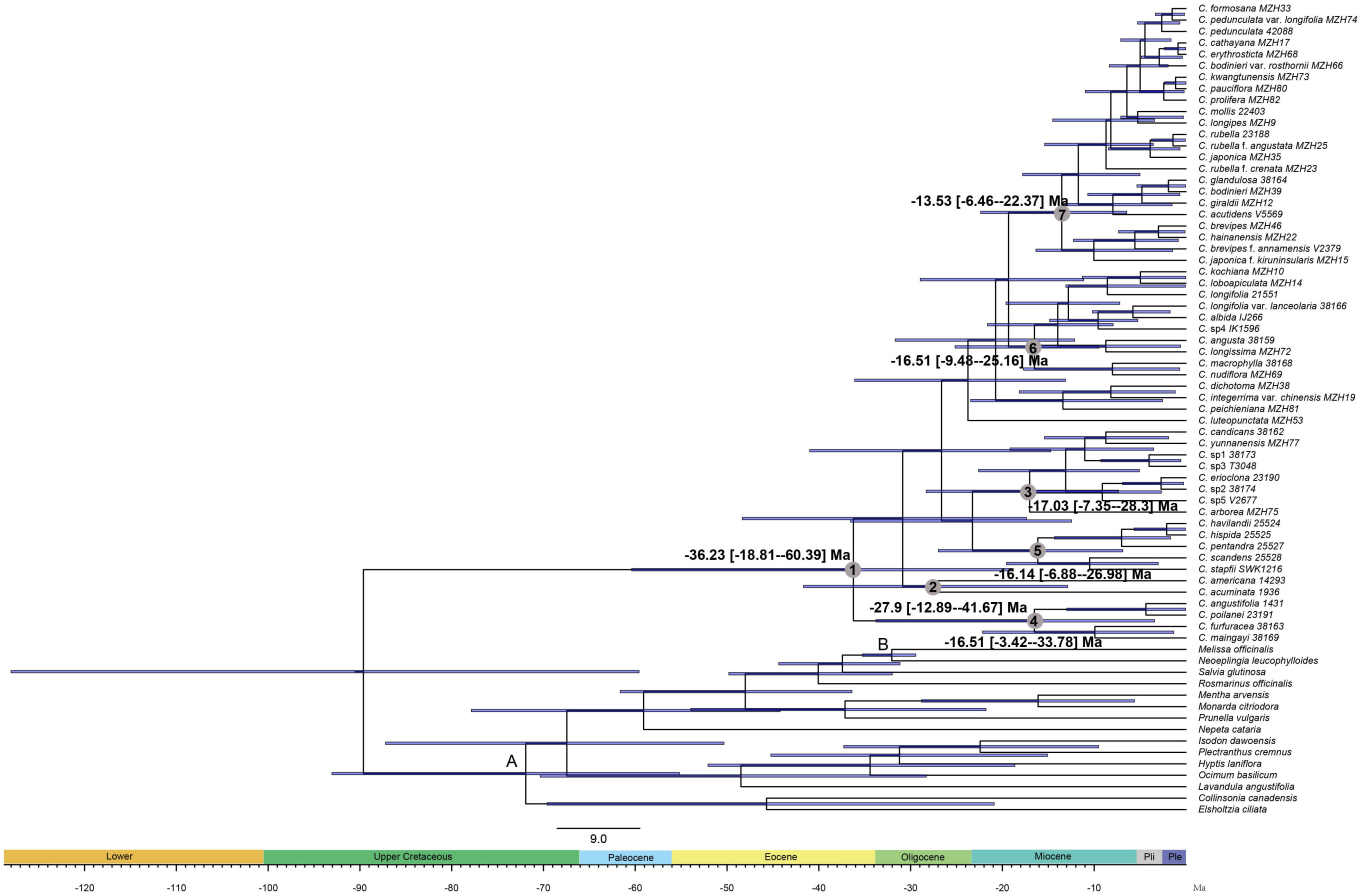
BEAST chronogram of *Callicarpa* inferred from combined DNA data (two nuclear and eight chloroplast regions) based on two fossil calibrations. Bars representing the 95% highest posterior density intervals. Node A was constrained to 49 Ma and node B was constrained to 28.4 Ma.

**Figure 3-1:**
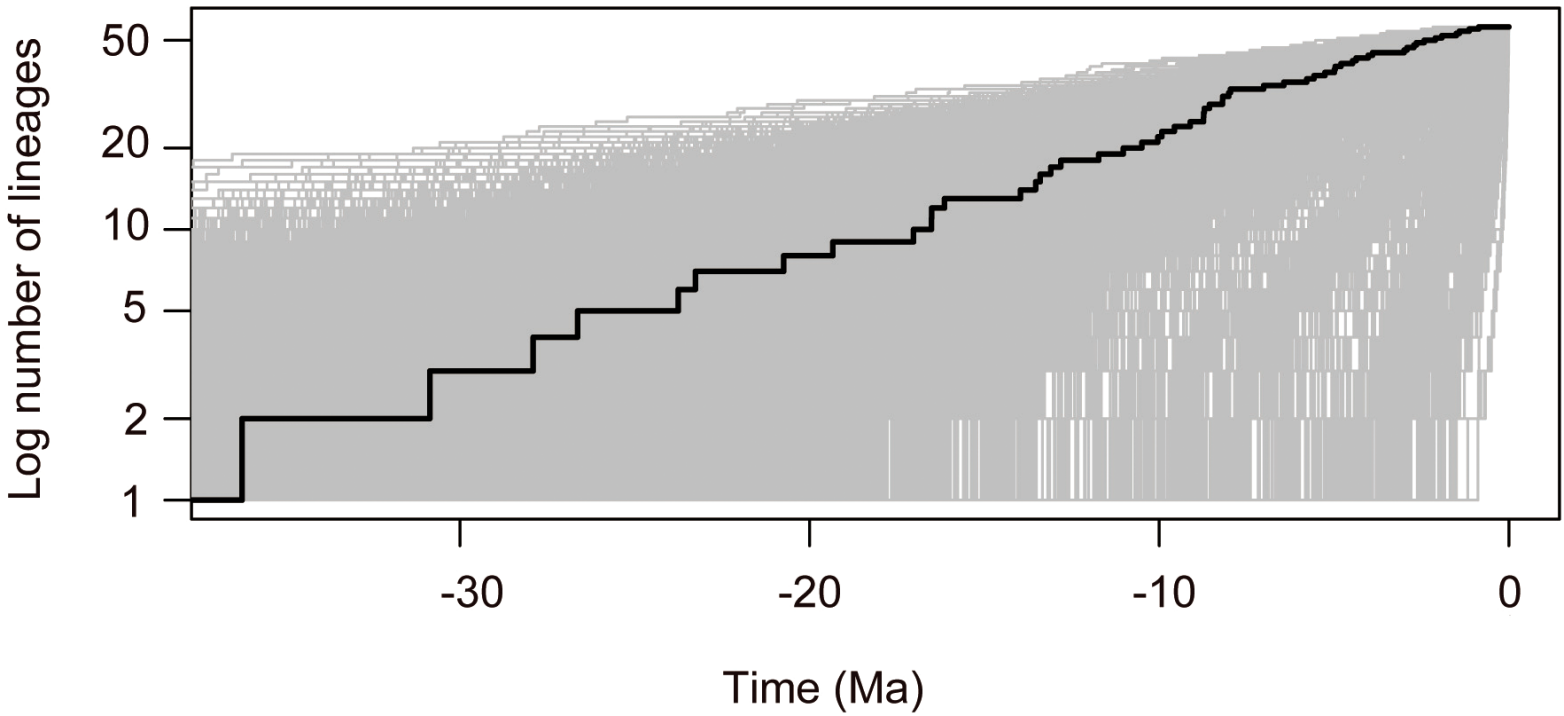
Standard LTT plots for *Callicarpa* clade are presented.

**Figure 4:**
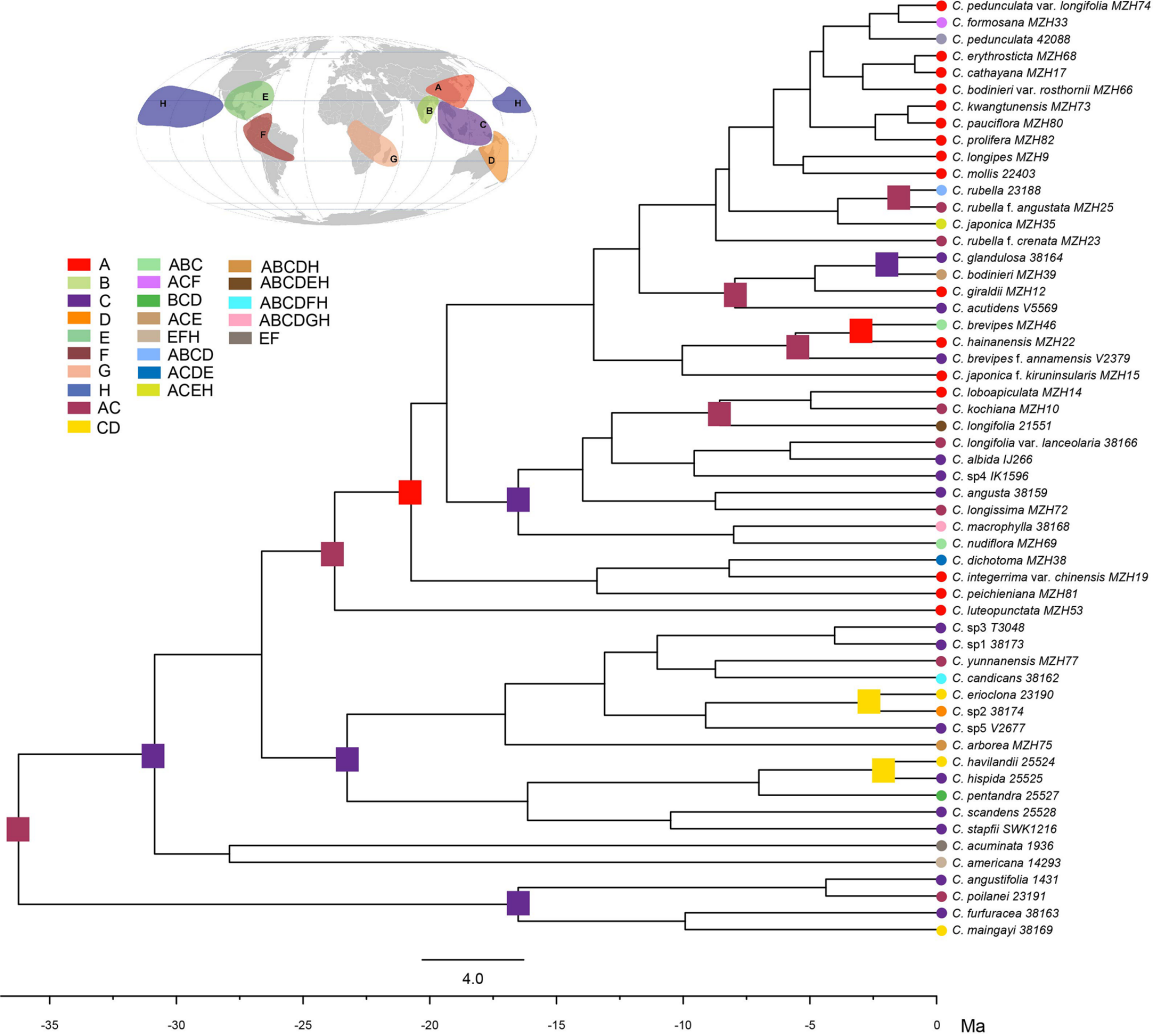
Ancestral range estimation of *Callicarpa* using the BEAST maximum clade credibility chronogram. Letters a-g indicate nodes of interest. Ancestral range reconstruction was performed by S-DEC in RASP. Map showing eight biogeographical regions in colors as defined in this study.

The statistical dispersal extinction cladogenesis (S-DEC) model reconstructed a composite area of both East Asia and Southeast Asia as the likely ancestral areas for the recent common ancestor (MRCA) of *Callicarpa* at approximately 36.23 Ma in the Late-Eocene (**Figure 4**, node A). There was one dispersal event from East Asia to Southeast Asia and one extinction event in East Asia. The early diversification of a *Callicarpa* ancestor occurred at 30.86 Ma (95% HPD: 17.37-48.34 Ma) and the first extinction event was inferred to have occurred in East Asia (**Figure 4**, node B). During this period, this genus may have undergone an eastward dispersal from Asia to Pacific Islands and subsequently there could be dispersal, vicariance, and extinction events resulting in the split between the Old World *Callicarpa* clade and the New World *Callicarpa* clade (**Figure 4**, node C). Subsequently, there was the first dispersal from Southeast Asia to East Asia (**Figure 4**, node D, *ca.* 26.63 Ma). During the early-middle Miocene, there were the first dispersal event which led to the successful colonization of the genus in Oceania (**Figure 4**, node F, *ca.* 17.03 Ma) and similar dispersal events also occurred after middle Miocene. The dispersal from Asia into the Mascarene Islands and Reunion Island took place at 8.01 Ma (95% HPD: 0.68-17.71 Ma) (**Figure 4**, node G). After several vicariance and extinction events, some species endemic to China arose. The diversification of *C. luteopunctata* occurred around 23.75 Ma (95% HPD: 13.12-36.09 Ma). *Callicarpa hainanensis*, a new species discovered from Hainan, China, diverged at 3 Ma (95% HPD: 0.1-7.35 Ma) with a vicariance event during the boundary period of the Miocene and Pliocene. The RASP analysis suggested that the ancestral area of these species was East Asia and Southeast Asia, and under the influence of these events they were only occurring in China or Southeast Asia. In summary, multiple dispersal and vicariance events have occurred over the evolutionary history of *Callicarpa*.

## 4 Discussion

### 4.1 Phylogenetic inference

**Figure 5:**
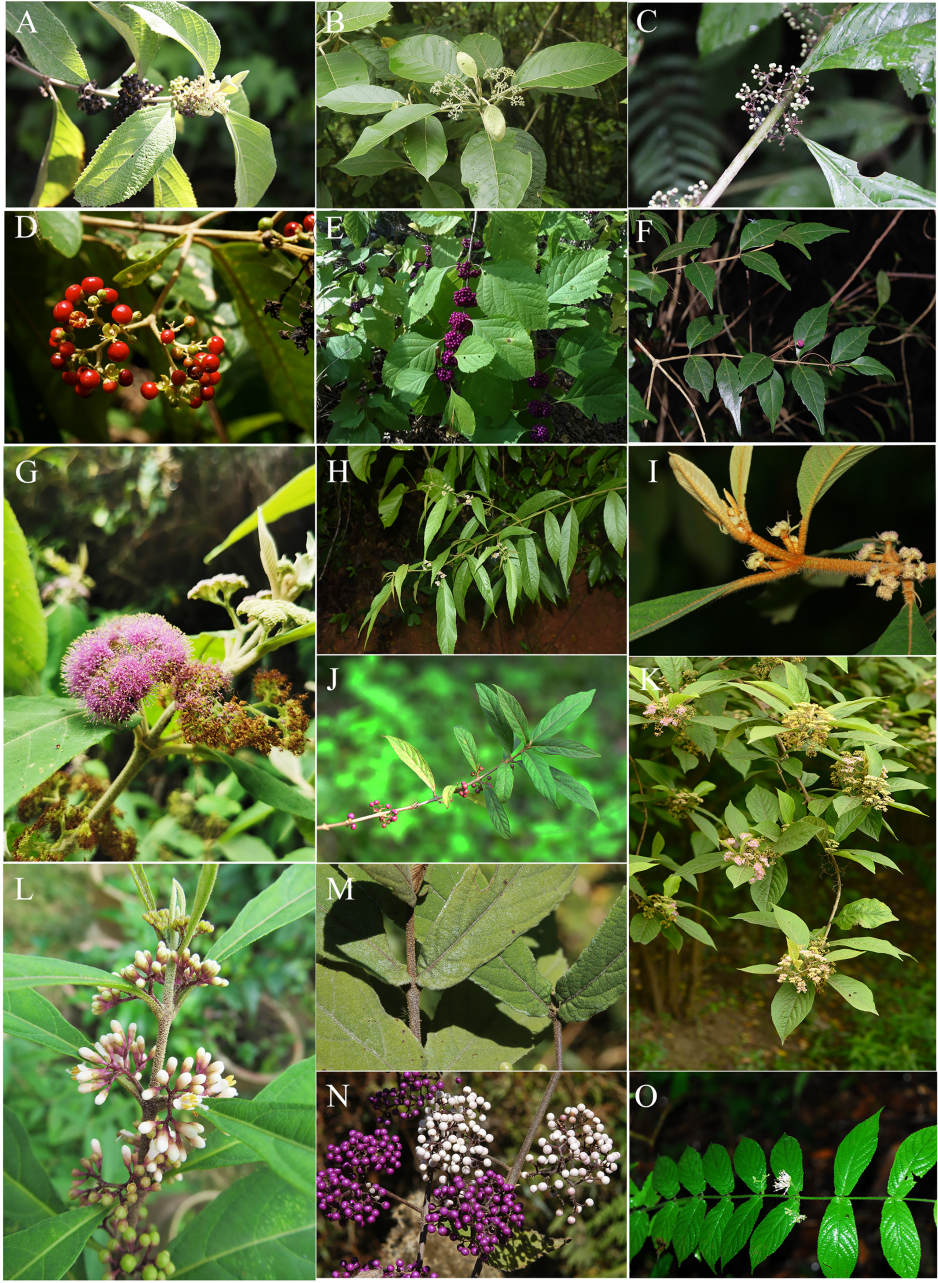
Morphological characteristics of *Callicarpa* for phylogenic discussion. **(A)** *C. candicans*; **(B)** *C. arborea*; **(C)** *C. luteopunctata*; **(D)** *C. pentandra*; **(E)** *C. americana*; **(F)** *C. peichieniana*; **(G)** *C. macrophylla*; **(H)** *C. longifolia*; **(I)** *C. kochiana*; **(J)** *C. brevipes*; **(K)** *C. bodinieri*; **(L)** *C. kwangtungensis*; **(M, N)** *C. rubella*; **(O)** *C. longipe*.

The combined analysis indicated that *Callicarpa* was monophyletic (PP=1.00, BP=100) with respect to the groups considered, and within *Callicarpa*, eight main subgroups were recognized (clade I-VIII) (**Figure 2**). Clade I, located at the bottom of the phylogenetic tree of *Callicarpa*, was composed of some Malaysian and Thai species: *C. poilanei*, *C. angustifolia*, *C. maingayi*, and *C. furfuracea*. After examining and comparing the type of specimens of *C. angustifolia* and *C. poilanei*, Leeratiwong et al. (2007) regarded *C. poilanei* as the synonym of *C. angustifolia* because they bear a resemblance in having a prominently interpetiolar woody ridge at the stem nodes, grey to brownish-grey hairs on the abaxial surface of leaves, and being glabrous or with sparsely hairy ovary. In the present study, our result supported Leeratiwong’s treatment (PP=1.00, BP=100). *Callicarpa furfuracea* and *C. maingayi* formed a robust clade, which suggested their closer relationship. Evidence from morphology (Leeratiwong et al., 2007; Leeratiwong et al., 2009) seemed to favor this clade. Clade II contained several small tree species, with tropical occurrences (Thailand, Vietnam, Indonesia, Malaysia, New Guinea), although *C. candicans*, *C. yunnanensis*, and *C. arborea* also occur in some narrow areas of southern China (south of Hainan Island and Xishuangbanna, Yunnan). In Bramley’s study (Bramley, 2009), *C. candicans* formed a clade with *C. furfuracea*, *C. maingayi*, *C. angustifolia*, and *C. poilanei* (assigned into section Clade I in our study) without support rate. However, in the present study, *C. candicans* exhibited a closer sister relationship with *C. erioclona* and *C. yunnanensis* (PP=1.00, BP=71). Morphologically *C. candicans* is most easily confused with *C. erioclona*, but the indumentums on the outer surface of the ovary and fruit may be effective morphological markers to distinguish them. In *C. erioclona*, the ovary and fruit are covered with branched hairs while that of *C. candicans* is glabrous. In addition, the black fruit (when mature) of *C. candicans* (**Figure 5A**) allows it to be distinguished from *C. erioclona* (maturing purple). Within clade II, *C. arborea* (**Figure 5B**) appeared at the top of the clade and as the sister of other tree species. Although *C. arborea* and *C. yunnanensis* are extremely similar and share an overlapped distribution, they appeared in different branches in clade II. Geographically, *C. arborea* is one of the most common species of the genus with a wide distribution (almost everywhere in Southeast Asia, Leeratiwong et al., 2009; Bramley, 2013), while Yunnan (Southwest China) is the edge of its northernmost distribution (Fang, 1982; Chen and Gilbert, 1994). Contrarily, *C. yunnanensis* is just narrowly distributed in the mixed forests of valleys in southern Yunnan and northern Vietnam (Fang, 1982; Chen and Gilbert, 1994). For palynology, two species can be distinguished from each other by their different types of pollen exine ornamentation: the pollen of *C. yunnanensis* has an exine sculpture of rugosely reticulate while *C. arborea* has regulate exine ornamentation (Ma et al., 2016). *Callicarpa luteopunctata* (**Figure 5C**) formed an independent branch (Clade III) and this species has an extremely narrow geographical distribution, only found in high altitude mountains of the Yunnan-Guizhou Plateau located in Southwest China. Their branchlets are cylindrical and there are no ridges or hair ring between the two petioles, and the peduncle is usually shorter than the petiole. In particular, both sides of the leaves have densely yellow glades. Clade IV contained several Indonesia-Malaysia-Thailand distributed species originally described as *Geunsia* (**Figure 5D**). Our results indicated that the ‘*Geunsia*’ group formed a well supported clade (PP=1.00, BP=100). The New World group was composed by *C. americana* (**Figure 5E**) and *C. acuminate*, which are ranging in North America and they formed a clade (Clade V, PP=0.89, BP=83). Old World and New World lineages also shows a complex phylogenetic relationship. In the present study, *C. integerrima* var. *chinensis* and *C. dichotoma* formed Clade VI, which represented the few lianas or slender-climbing species of *Callicarpa* mainly distributed in China (**Figures 1B, E**). When carrying out a palynology study on *Callicarpa* in China, Ma et al. (2016) suggested that the distinct coarsely reticulate exine sculpture was found only in certain species – those that represented the climbing shrubs species of *Callicarpa* native to China (*C. integerrima*, *C. integerrima* var. *chinensis* and *C. pilosissima*). Based on the distinct, strongly curved hairs on the stem and extremely simple cymes (only 1–3 flowers, or one dichotomous, **Figure 5F**), Fang (1982) treated *C. peichieniana* as a monotypic subgenus (Subgen. *Peiantha* Chun et S. L. Chen) and other species constituted the subgenus *Callicarpa*. However, our phylogenetic result from ML analyses showed that *C. peichieniana* was mixed with other species (Subgen. *Callicarpa*), which does not support Fang’s classification system (Fang, 1982). The traditional subgenus classification system of *Callicarpa* based on only two characters is unpredictable. Clade VII was further divided into two main subclades: subclade VII_I_ and subclade VII_II_. Subclade VII_I_ consisted of *C. nudiflora* and *C. macrophylla* (**Figure 1D**; **Figure 5G**), and both species share a series of common characteristics. However, inflorescence width and peduncle length are different between the two species, while the most significant difference to distinguish the two species is that the calyx, corolla, and ovary of *C. macrophylla* are covered by stellate tomentose, while those of *C. nudiflora* are glabrous. In addition, it is worth bearing in mind that the leaves of *C. nudiflora* turn black after being dried while those of *C. macrophylla* do not. Subclade VII_II_ contained several species characterized by a conspicuous interpetiolar ridge resembling a stipule scar, and a white baccate drupe with an obviously softer fleshy exocarp than other species of *Callicarpa*. Yet the color and textures of the exocarp have never attracted the attention of previous taxonomic researchers working on *Callicarpa*. In Subclade VII_II_ (PP=0.98, BP=97), these species are primarily distributed in China, although *C. longifolia* is considered as a broadly distributed species (**Figure 5H**), and *C. angusta* is endemic in Vietnam. Based on *C. kochiana* tubular calyx (**Figure 5I**), Fang (1982) assigned *C. kochiana* as a monotypic section *Tubulosae*, and divided other species of the subgenus *Callicarpa* into Section *Callicarpa*. In the present study, *C. kochiana* is embedded in *C. longifolia* and *C. loboapiculata*, and together form a clade (PP=0.43, BP=56), which shares a series of synapomorphy: a conspicuous interpetiolar ridge and white baccate drupe. Increasing the number of samples of *C. kochiana* and more genetic data is all needed to confirm its species status (Chanderbali et al., 2001; Small et al., 2004; Guo and Ge, 2005; Liu et al., 2021). Clade VIII could be recognized as one broader clade which was strongly supported as the largest group of *Callicarpa* investigated in the present study (PP=1.00, BP=100), and comprised of some taxa with a broad range of morphological variation. The Subclade VIII_I_ was formed with good support (PP=1.00, BP=100). Among them, *C. hainanensis* and *C. brevipes* formed a small clade (**Figures S2-1, S2-2**, PP=0.96, BP=84) characterized by their lanceolate or obovate-lanceolate leaves (**Figure 5J**), and the two species share a series of typical characters of Chang’s (1951) section *Verticirima*, such as larger, oblong, apical pore dehiscent anthers and short filaments. However, *C. hainanensis* is obviously distinguished from the latter by its obovate-lanceolate leaves, long-cup-shaped or subtubular calyx dehisced as the fruits mature, and sharp triangular lobes (Ma and Zhang, 2012). *Callicarpa bodinieri* is easily distinguished by its red subsessile glands on stem, leaves, and flowers from other species (**Figure 5K**), and Leeratiwong et al. (2009) found that *C. bodinieri* and *C. glandulosa* are conspecific by examining their specimens. Therefore, Leeratiwong et al. (2009) reduced *C. glandulosa* to be a synonym of *C. bodinieri.* In this study, they also formed a very close relationship (**Figures S2-1, S2-2**, PP=1.00, BP=92). Within subclade VIII_II_, *C. pedunculata*, *C. formosana*, and *C. pedunculata* var. *longifolia* clustered into one subclade (**Figures S2-1, S2-2**, PP=1.00, BP=100). During the taxonomic revision of Philippines *Callicarpa*, Bramley (2013) treated *C. formosana* as one of the numerous synonyms of *C. pedunculata*, a species with an extensive distribution and significant variation in leaf shape. As our molecular results suggest, *C. pedunculata* from Australia was nested within *C. formosana*, so we supported reduction of *C. formosana* to the synonym of *C. pedunculata* according to the rules of nomenclature. Bramley (2013) indicated that *C. pedunculata* was most likely to be confused with *C. rubella*, from which it differed by its typically relatively narrow leaves and lack of glandular hairs but with abaxially stellate tomentose, adaxially minute hispid, and an obtuse or rounded base. Their close relationship was also verified in the present phylogenetic study as two species appearing in close sister clades. It was unexpected that *C. kwangtungensis* (**Figure 5L**), designated as a typical species of section *Verticirima* in the Chang (1951) system, formed a sister clade to *C. prolifera* and *C. pauciflora* (**Figures S2-1, S2-2**, PP=0.95, BP=99). For *C. rubella*, morphologically, we found in our field investigation that white fruit populations of *C. rubella* always mix with purple fruit populations in the coinhabiting areas (**Figures 5M, N**). As a broadly distributed species, *C. rubella* is variable morphologically, especially in terms of its indumentum and the size and shape of the leaves. The significant difficulty in determining the identity of the complex group of *C. rubella* based on morphology and phylogenetic placement suggests this group may potentially represent hybrids, although we are unaware of the specific parental origin. It makes sense that identical repeated interspecies hybridization may occur in *C. rubella* and its infraspecific taxa. In Japanese *Callicarpa*, Tsukaya et al. (2003) reported the hybridization and introgression occurring between *C. japonica* and *C. mollis* in central Japan with molecular data, observing that both species were pollinated by bees (Kawakubo, 1990; Momose et al., 1998; Kato et al., 1999; Kato, 2000). Xu et al. (2013) investigated the cross breeding between *C. dichotoma* and *C. bodinieri* in China and their result indicated that there was no crossing barrier between the two species, and they observed a high fruit setting ratio. *Callicarpa longipes* and the complex group of *C. rubella* formed a group morphologically well delimited with other species by a cordate leaf base (**Figures S2-1, S2-2**, PP=0.73, BP=94) (**Figure 5O**). The complex group of *C. rubella* has troubled researchers for a long time in terms of distinguishing each species due to their extremely ambiguous morphological circumscription. We have to pay attention to *C. japonica* which formed a close relationship with *C. rubella* f. *crenata* (**Figures S2-1, S2-2**, BP=100, PP=1.00). In Bramley’s revision of Bornean *Callicarpa* (Bramley, 2009), the sister clade of *C. japonica* and *C. rubella* was also represented.

### 4.2 East Asia and Southeast Asia as the ancestral area and *Callicarpa*’s lineage diversification within Asia

The ancestral range reconstruction analyses indicated that *Callicarpa* most likely originated in East Asia and Southeast Asia (**Figure 4**, node A). With our time estimates, the S-DEC model inferred that the main diversification events in *Callicarpa* were dated to the Middle-Oligocene, mainly concentrated to the Miocene (**Figure 4**). Multiple dispersal events were likely responsible for the current biogeographic patterns of the genus. This biome began to rise in the early Miocene (*ca.* 20 Ma) and further diversified in the late Miocene, driven probably by the intensifying East Asian summer monsoon during these two periods (Guo et al., 2002; Sun and Wang, 2005). In later branching lineages the genus might have experienced more rapid diversification in the Middle-Miocene (**Figure 3-1**, LTT plot). It probably corresponded with one of the major uplifts of the QTP and subsequent aridification events (Yu et al., 2014). Before about 14 Ma, there was a relatively stable diversification rate and subsequently an accelerated lineage accumulation. This upward trend was maintained during the Pliocene and the Pleistocene, which had been attributed to topographic and climatic circumstances (Qian and Ricklefs, 2000). Since the middle-late Oligocene to the Miocene, the coverage of Antarctic glaciers shrunk owing to global warming, and there was a high point of temperature in the middle-Miocene (Gao et al., 2020). Donoghue and Smith (2004) once proposed that the Miocene was one of the active periods of species diversification. The occurrence of the monsoon was characterized by changes in the prevailing wind direction and severe precipitation. It was often accompanied by the rapid strengthening of atmospheric-energy and the water cycle, which directly affected the global hydrothermal cycle and heat distribution, and regulated global climate change (Webster et al., 1998; An et al., 2000; Jiang et al., 2017). Numerous studies have suggested that Southeast Asia is a ‘museum’ of early angiosperms, harboring tropical rain forests with the most pronounced monsoon climate and acting as an ‘evolutionary front’ for some tropical taxa, given that it has the largest archipelagos and probably has the most complex geological history in the world (Van Welzen et al., 2011; Tan et al., 2020). The extremely rich biodiversity in Southeast Asia hints not only to one of the birthplaces and refuges of early angiosperms (Buerki et al., 2014), but also to a meeting point for the long-distance spread of species (Gunasekara, 2004; Lohman et al., 2011), which profoundly affects the formation and evolution of global flora. The Sunxdaland (Malay Peninsula; Borneo; Sumatra) and the Philippines are two acknowledged biodiversity hotspots in Southeast Asia (Myers et al., 2000) and there appear to be two major centers of diversity in terms of numbers of Malesian species for *Callicarpa*: Borneo and the Philippines (Bramley, 2013). Moreover, our findings likewise suggested that East Asia and Southeast Asia are main sources of biodiversity of *Callicarpa*.

**Figure 6:**
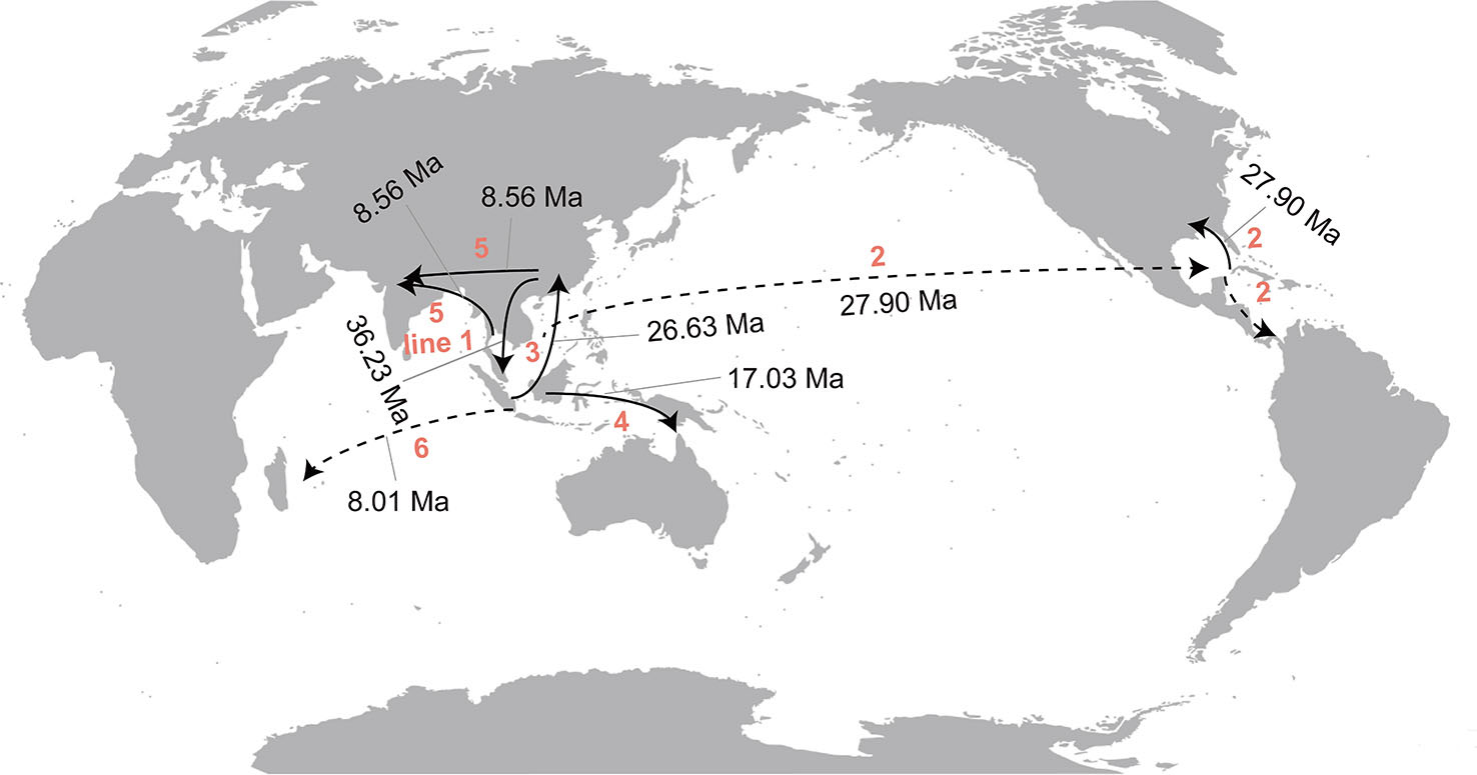
Historical migrations in Callicarpa estimated from the ancestral area reconstruction. The estimated times for the first migration among areas are shown. The dotted line represents the guessed route according to the distribution of the genus.

Around 23.75 Ma (95%HPD: 13.12-36.09 Ma), there occurred the third extinction event in Southeast Asia resulting in *Callicarpa luteopunctata* splitting from the remaining species (**Figure 4**, node E). This species has an extremely narrow geographical distribution and is found only in high altitude mountains of the Yunnan-Guizhou Plateau located in Southwest China. Coincidentally, *C. kinabaluensis* and *C. clemensorum* were also reported to be narrowly distributed at high altitude (1600–2500 m) on the peaks surrounding Mount Kinabalu in Malesia (Bramley, 2011). A set of common features shared by *C. kinabaluensis* and *C. clemensorum* was speculated to be an adaptation to the frigid environment: so dense hairs and an interesting inflorescence structure with distinct peduncles and almost globose cymes. In addition, their twigs have scattered warty growth and the lamina surface occurred vesiculose (Bramley, 2009). However, *C. luteopunctata* has the indumentum on the petioles and cymes obviously sparser than that of *C. kinabaluensis* and *C. clemensorum*. It is a pity that this study failed to contain the two Malaysian species and we still lack an understanding of the unambiguous mechanism of these specific high altitude species in the genus *Callicarpa* of typical near tropics originated (Bramley, 2009; Bramley, 2013; Ma et al., 2016). Further studies on how the mechanism developed are urgently needed in the future. However, the onset of the Asian monsoon around the Oligocene-Miocene transition created a connection between forests from the low to high latitudes of East Asia (Sun and Wang, 2005; Ji et al., 2019), which might provide some insights. Subsequently, more frequent exchanges took place in the Asian interior among species we investigated in the present study. At 36.23 Ma (95%HPD: 18.81-60.39 Ma) there was the first dispersal from East Asia to Southeast Asia (**Figure 4**, node A; **Figure 6**, line 1) and subsequently, after the Oligocene, several similar dispersals (from East Asia to Southeast Asia) happened again with higher probability in *Callicarpa*. At the Oligocene-Miocene boundary, the Tibetan Plateau experienced a rapid uplift and previous geological evidence indicated that different areas of the Plateau have experienced different degrees of uplift at different times (Neogene and Quatemary) (Royden et al., 2008; Wang, 2017). These uplifts since the early Miocene have created high mountains and deep valleys within the plateau, which could have accelerated the production of new allopatric species, and been partly responsible for the high local and regional species richness (Liu et al., 2006) and induced extreme drying and desertification in the Asian interior, strengthening the Asian monsoons with a shift occurring from arid/semi-arid in the Asian continental interior (Guo et al., 2002; Sun and Wang, 2005). These factors probably effected the expansion of *Callicarpa* species within Asia regions later on (**Figure 6**, line 3, 5). Our study indicated that at 26.63 Ma (95%HPD: 14.72-41 Ma), there was first migration from Southeast Asia to East Asia (**Figure 4**, node D **Figure 6**, line 3) and during the Miocene-Pliocene boundary, two vicariance events occurred between Southeast Asia and East Asia, which influenced *C. brevipes* f. *kiruninsularis*, *C. hainanensis*, and *C. giraldii* all endemic to China. Under the influence of tectonic motion and climate fluctuation, dispersal and vicariant events occurred alternately, and habitats periodically isolated and merged, which may accelerate species differentiation in this process (Thomas et al., 2012). In Asia, East Asian monsoons, South Asian monsoons, and Northwest Pacific monsoons prevailed in summer (Jiang et al., 2017), which might have promoted the rapid differentiation and spread of *Callicarpa* and facilitate tropical species to spread northward (mainly to the area now China) by regulating rainfall. Apparently, the climate of Asia was controlled mainly by the monsoon system due to intense land-ocean thermal contrast, and the dynamics and thermal effects of the Tibet Plateau (Sun and Wang, 2005). The monsoon climate intensified from the late-Miocene, simultaneously bringing an East Asian subtropical humid climate and promoting the floristic expansion of Asia (Ji et al., 2019). We speculated that climatic change and global cooling since the mid-Miocene might have played a crucial role in the inferred onset of diversification of *Callicarpa* in Asia. During this time, several independent vicariance events between East Asia and Southeast Asia occurred, which accelerated some endemic species to form in China. Also, the S-DEC model revealed the divergence of *C. dichotoma* and *C. japonica*, two species distributed in Japan. The divergence times of them were 8.19 Ma (Miocene) and 3.9 Ma (Pliocene), and our results speculated that the opening of the Japan Sea corresponded roughly to their spread from continental East Asia to the Japan islands (Santosh and Senshu, 2011; Ji et al., 2019). Sea levels fluctuating alternately enabled alternate conditions for population fragmentation and admixture of temperate biota in this East China-Japan-Korea region (Qiu et al., 2011). It has long been recognized that the climatic changes of the Quaternary caused repeated shifts in the distribution of plants and animals presently found throughout the SJFR (Sino-Japanese Floristic Region). The RASP analysis also suggested the last ancestral area of *C. giraldii* was East Asia and Southeast Asia, and under the influence of one vicariance event, the species occurred only in China. *C. giraldii* seems able to tolerate a relatively cool climate and is widely distributed from subtropical Southeast China (Jiangxi, Fujian, Hunan) to temperate areas (Henan, Shanxi). Morphologically, they are small shrubs with cabined, tight cyme.

### 4.3 The disjunct pattern between the Paleotropics and Neotropics in *Callicarpa*

*Callicarpa* also presents an amphi-Pacific distribution pattern. Our results revealed that during the Middle-Oligocene, floristic exchange in *Callicarpa* occurred between Southeast Asia and the Temperate North America-Neotropical region (**Figure 4**, node C). Nevertheless, our inferred date for migrations of *Callicarpa* from the Paleotropics to Neotropics is too young to have been caused by the breakup of Gondwana (Lomolino, 2010; Jin et al., 2020). The oft-stated view is that the North Atlantic Land Bridge (NALB) or Bering Land Bridge (BLB) has played a major role in this process (Wolfe, 1975; Tiffney, 1985; Tiffney and Manchester, 2001; Chen et al., 2020; Zhang et al., 2020b). The NALB generally functioned as a famous migration route for thermophilous plant taxa between Europe and eastern North America (Tiffney and Manchester, 2001; Ian, 2006; Jin et al., 2020). Tiffney and Manchester (2001) summarized biogeographic continuity across the North Atlantic Ocean during the Tertiary and pointed out that the bridge was broken by late Eocene. According to the lack of records of the extant or fossil species of *Callicarpa* from Europe, the route (by the NALB) seems infrequent for this originally tropical genus and it is also believable that the NALB was no longer available at the time for the dispersal of *Callicarpa*. Likewise, the way *via* Beringian connections seems unlikely owing to the mostly tropical affinities of *Callicarpa* (Chen et al., 2020; Li et al., 2020a). To explain this amphi-Pacific tropical and subtropical disjunction occurring in *Callicarpa*, the ‘Long-distance migration across the Pacific Ocean’ route was speculated herein, combined with our S-DEC inferences. Yang et al. (2018) reported that *Kingsboroughia alba* (Sabiaceae), with an amphi-Pacific tropical disjunct distribution, migrated from Central America to tropical Asia *via* long-distance dispersal during the time of the Neogene and Quaternary boundary. Similar transoceanic biogeographical patterns have been found in other plant groups, such as Nettles (Urticeae, Urticaceae) (Huang et al., 2019), Lardizabalaceae (Wang et al., 2020), and polystichoid ferns (Dryopteridaceae) (Le Pechon et al., 2016). In our results, owing to the taxa discovered from Western Pacific Islands and Hawaiian islands, a hypothesis could be proposed that during the Middle-Oligocene the ancestor of *C. americana* and *C. acuminate* dispersed for the first time from Southeast Asia to the Neotropics by trans-ocean routes (**Figure 6**, line 2) and subsequently further migrated northward to temperate North America and southward to South America (**Figure 6**, line 2), resulting in the amphi-Pacific disjunction pattern of the genus *Callicarpa*. The achievement of plant migration between North America and South America was also partly related to taking advantage of island stepping-stones (island hopping) and stochastic long-distance dispersals (Gentry, 1982; Bacon et al., 2015). In late Tertiary, the colonization of *Callicarpa* in Pacific Islands, Temperate North America, and the Neotropics occurred again. Physical connections between affected areas provide inference about the biogeographic history. The uplift of the northern Andes and the rising of volcanic islands may have provided the conditions responsible for the exchange of *Callicarpa* between Central and South America (Li and Wen, 2013), and these islands eventually coalesced into today’s lower Central America with a substantial land connection across the Isthmus of Panama (Gentry, 1982). The Panama Isthmus as the narrow strip of land connected North and South America and offered a channel for migration in the Pliocene after it closed (Keigwin, 1978). In the New World, 33 species of *Callicarpa* were recognized, particularly on the Caribbean Islands (24 species currently recognized in Cuba) (Bramley, 2013). Cuba possibly has broadly similar climates and floristic components to Southeast Asia (they are at similar latitudes) (Milne and Abbott, 2002) and Cuba appears to be another center of diversification of *Callicarpa*. In sum, a trans-Pacific dispersal between two regions is plausible (Wang et al., 2020) to explain the establishment of a New World distribution of *Callicarpa*, and this hypothesis requires further analysis and testing. *Callicarpa* successfully reached the Hawaii Islands, other oceanic islands and the Neotropics, to some extent reflecting efficient seed dispersal by birds, usually attracted by small and brightly colorful fruits (especially purple fruits) (Popp et al., 2011). Interestingly, in the progress of tracking the extraordinary migratory journeys and broad-scale habitat use of sooty shearwaters, Shaffer et al. (2006) found that this small seabird can fly right across the Pacific Ocean. Biotic seed dispersal is possibly correlated to large species range sizes and most biotically dispersed species with colorful berries (fleshy fruit) and endozoochorous seeds embedded in juicy pericarp were regarded more suitable for frugivorous birds to spread over long-distance (Loiselle and Blake, 1999; Kessler-Rios and Kattan, 2012; Tan et al., 2020). When the mode of dispersal of *Cornus* was studied, Lindelof et al. (2020) reported that ‘Island hopping’ was a possible mode of dispersal in bird-dispersed genera (producing fleshy, nutritious fruits). However, what kinds of birds are involved in the spreading of *Callicarpa* is still unknown, and further studies about dispersal agents and other driving factors are required to gain more insights into the patterns of diversification of *Callicarpa*.

### 4.4 Migrations between Southeast Asia and oceanic regions or islands of the Indian ocean

During the early middle-Miocene, population exchanges of *Callicarpa* among Southeast Asia, Papua New Guinea, and Northern Australia may have occurred (**Figure 6**, line 4). Between the regions of East and Southeast Asia and Australasia multiple migrations of *Callicarpa* occurred during different geological periods (the Neogene and Quatemary). The Australian plate started to move northward at the beginning of the Paleogene, *ca.* 45 Ma and Northern Australia reached the tropics by the beginning of the Miocene, progressively moving northwards each year subsequently (Li and Powell, 2001; Yuan et al., 2005), colliding with the Southeast Asian plate in the middle-Miocene (Lee and Lawver, 1995; Tan et al., 2020), which quite possibly facilitated the flora exchanges among these regions. Furthermore, the onset of the New Guinean highland orogenesis in the late Miocene (Zachos et al., 2001) and putative island chain connections between Asia and Australia during mid to late Miocene (Baldwin et al., 2012; Wu et al., 2019; Li et al., 2020b) made migrations possible. The emergence of the land masses of the eastern Wallace’s Line including Wallacea, Sulawesi, New Guinea, and a series of volcanic islands along the Sunda Arc, the Banda Arc, and the Halmahera Arc connecting these regions from the late Miocene onwards, probably offered a potential channel for dispersals between the two regions (Hall, 2009). Whereafter, the declining sea level during the maximum glacial period (Hantoro et al., 1995; Tan et al., 2020) undoubtedly provided more chances for the exchange of *Callicarpa*, including its spread westward —the opposite dispersal from northern Australia and the island of New Guinea to Southeast Asia. The last common ancestor of *C. macrophylla* has experienced one westward dispersal to the Mascarene Islands and Reunion Islands (**Figure 6**, line 6). Molecular dating frames for the African-Asian disjunction ranged from the Cretaceous (Conti et al., 2002) to the Pleistocene (Li et al., 2009). Our divergence time estimates for *Callicarpa* appear too young to be explained by Indian rafting (Cretaceous). Researchers have built a strong argument that many tropical taxa could have migrated between Africa and Asia through Arabia (Zhou et al., 2012; Jin et al., 2020). However, this overland migration does not seem likely because there are no records of extant species of *Callicarpa* from Arabia. The best candidate for explaining the dispersal from Southeast Asia to Madagascar might be through transoceanic long distance dispersal (Les et al., 2003; Yuan et al., 2005; Yao et al., 2016).

As a consequence, this diversity is not evenly distributed and fairly closely follows the distribution of certain topographic and climatic conditions. Major biogeographic lines were crossed frequently and multiple past colonization events had left traces.

## 5 Conclusions

Our phylogenetic results indicated that *Callicarpa* is monophyletic with respect to the groups considered, and eight primary clades were well supported. We supported a two-species treatment of certain synonyms, regarding *C. poilanei* as the synonym of *C. angustifolia* and *C. formosana* as the synonym of *C. pedunculata*. Our biogeographic analyses suggested that the probable ancestor of the *Callicarpa* crown clade originated in the tropical regions of East Asia and Southeast Asia around the Late-Eocene. The early diversification of a *Callicarpa* ancestor occurred at 30.86 Ma and during that period this genus may have undergone an eastward dispersal from Asia to Pacific Islands by trans-ocean long-distance dispersal. Subsequently, there could be dispersal, vicariance, and extinction events resulting in the split between the Old World *Callicarpa* clade and the New World *Callicarpa* clade. Subsequently, Asian summer monsoons probably contributed to the diversification of the *Callicarpa* lineage within Asia. Around the early middle-Miocene, the onset of the New Guinean highland orogenesis and putative island chain connections made migrations to Oceania possible. The dispersal from Southeast Asia to Madagascar might have been achieved through transoceanic long distance dispersal.

## Data availability statement

The datasets presented in this study can be found in online repositories. The names of the repository/repositories and accession number(s) can be found in the article/**Supplementary Material**.

## Author contributions

ZM and HC designed the study. HC analyzed the data and wrote the manuscript. XL collected the geographical data of species and WW performed the experiments. ZM, BL and GB collected plant material and provided valuable advices for the manuscript. All authors revised the manuscript and approved the submitted version.

## References

[B1] An Z. S. Porter S. C. Kutzbach J. E. Wu X. H. Wang S. M. Liu X. D.. (2000). Asynchronous Holocene optimum of the East Asian monsoon. Quaternary Sci. Rev. 19, 743–762. doi: 10.1016/S0277-3791(99)00031-1

[B2] Andersson S. (2006). On the phylogeny of the genus *Calceolaria* (Calceolariaceae) as inferred from ITS and plastid matK sequences. Taxon 55, 125–137. doi: 10.2307/25065534

[B3] Bacon C. D. Silvestro D. Jaramillo C. Smith B. T. Chakrabarty P. Antonelli A. (2015). Biological evidence supports an early and complex emergence of the isthmus of Panama. Proc. Natl. Acad. Sci. United States America 112, E3631–E3631. doi: 10.1073/pnas.1423853112 PMC4434730 25918375

[B4] Baldwin S. L. Fitzgerald P. G. Webb L. E. (2012). Tectonics of the new Guinea region. Annu. Rev. Earth Planetary Sci. 40, 495–520. doi: 10.1146/annurev-earth-040809-152540

[B5] Baldwin B. G. Markos S. (1998). Phylogenetic utility of the external transcribed spacer (ETS) of 18S-26S rDNA: congruence of ETS and ITS trees of *Calycadenia* (Compositae). Mol. Phylogenet. Evol. 10, 449–463. doi: 10.1006/mpev.1998.0545 10051397

[B6] Beardsley P. M. Olmstead R. (2002). Redefining phrymaceae: the placement of *Mimulus*, tribe mimuleae, and *Phryma* . Am. J. Bot. 89, 1093–1102. doi: 10.3732/ajb.89.7.1093 21665709

[B7] Bouckaert R. Heled J. Kuhnert D. Vaughan T. Wu C. H. Xie D.. (2014). BEAST 2: a software platform for bayesian evolutionary analysis. PloS Comput. Biol. 10, 6. doi: 10.1371/journal.pcbi.1003537 PMC3985171 24722319

[B8] Bramley G. L. C. (2009). The genus *Callicarpa* (Lamiaceae) on Borneo. Botanical J. Linn. Soc. 159, 416–455. doi: 10.1111/j.1095-8339.2009.00907.x

[B9] Bramley G. L. C. (2011). Distribution patterns in malesian *Callicarpa* (Lamiaceae). Gardens' Bull. Singapore 63, 287–298. Available at: http://biostor.org/reference/140290

[B10] Bramley G. L. C. (2013). The genus *Callicarpa* (Lamiaceae) in the Philippines. Kew Bull. 68, 369–418. doi: 10.1007/s12225-013-9456-y

[B11] Buerki S. Forest F. Alvarez N. (2014). Proto-South-East Asia as a trigger of early angiosperm diversification. Botanical J. Linn. Soc. 174, 326–333. doi: 10.1111/boj.12129

[B12] Chanderbali A. S. van der Werff H. Renner S. S. (2001). Phylogeny and historical biogeography of lauraceae: evidence from the chloroplast and nuclear genomes. Ann. Missouri Botanical Garden 88, 104–134. doi: 10.2307/2666133

[B13] Chang H. (1951). A review of the Chinese species of *Callicarpa* . J. Systematics Evol. 1, 269–312.

[B14] Chen S. Gilbert M. (1994). Verbenaceae. Flora China 17, 1–49.

[B15] Chen X. H. Xiang K. L. Lian L. Peng H. W. Erst A. S. Xiang X. G.. (2020). Biogeographic diversification of *Mahonia* (Berberidaceae): implications for the origin and evolution of East Asian subtropical evergreen broadleaved forests. Mol. Phylogenet. Evol. 151, 11. doi: 10.1016/j.ympev.2020.106910 32702526

[B16] Conti E. Eriksson T. Schonenberger J. Sytsma K. J. Baum D. A. (2002). Early tertiary out-of-India dispersal of crypteroniaceae: evidence from phylogeny and molecular dating. Evolution 56, 1931–1942. doi: 10.1111/j.0014-3820.2002.tb00119.x 12449480

[B17] Demesure B. Sodzi N. Petit R. J. (1995). A set of universal primers for amplification of polymorphic non-coding regions of mitochondrial and chloroplast DNA in plants. Mol. Ecol. 4, 129–134. doi: 10.1111/j.1365-294X.1995.tb00201.x 7711952

[B18] Dong W. Liu J. Yu J. Wang L. Zhou S. (2012). Highly variable chloroplast markers for evaluating plant phylogeny at low taxonomic levels and for DNA barcoding. PloS One 7, e35071. doi: 10.1371/journal.pone.0035071 22511980 PMC3325284

[B19] Donoghue M. J. Smith S. A. (2004). Patterns in the assembly of temperate forests around the northern hemisphere. Philos. Trans. R. Soc. B-Biological Sci. 359, 1633–1644. doi: 10.1098/rstb.2004.1538 PMC1693435 15519978

[B20] Doyle J. J. Doyle J. L. (1987). A rapid DNA isolation procedure for small quantities of fresh leaf tissue. Phytochemical Bull. 19, 11–15.

[B21] Drew B. T. Sytsma K. J. (2012). Phylogenetics, biogeography and staminal evolution in the tribe mentheae (Lamiaceae). Am. J. Bot. 99, 933–953. doi: 10.3732/ajb.1100549 22539517

[B22] Fang W. (1982). *Callicarpa* l. (Verbenaceae). Flora Reipublicae Popularis Sinicae 65, 24–79.

[B23] Fritsch P. W. Manchester S. R. Stone R. D. Cruz B. C. Almeda F. (2015). Northern hemisphere origins of the amphi-pacific tropical plant family symplocaceae. J. Biogeography 42, 891–901. doi: 10.1111/jbi.12442

[B24] Gao J. Yu T. Li J. (2020). Phylogenetic and biogeographic study of *Acer* (Sapindaceae) based on three chloroplast DNA fragment sequences. Acta Ecologica Sin. 40, 5992–6000. doi: 10.1038/s41598-020-78145-0

[B25] Gaynor M. L. Fu C. N. Gao L. M. Lu L. M. Soltis D. E. Soltis P. S. (2020). Biogeography and ecological niche evolution in diapensiaceae inferred from phylogenetic analysis. J. Systematics Evol. 58, 646–662. doi: 10.1111/jse.12646

[B26] Gentry A. H. (1982). Neotropical Floristic diversity: phytogeographical connections between central and south America, pleistocene climatic fluctuations, or an accident of the Andean orogeny? Ann. Missouri Botanical garden 69, 557–593. doi: 10.2307/2399084

[B27] Gunasekara N. (2004). Phylogenetic and molecular dating analyses of the tropical tree family dipterocarpaceae based on chloroplast matK nucleotide sequence data. Masters thesis. (Montreal: Concordia University).

[B28] Guo Y. L. Ge S. (2005). Molecular phylogeny of oryzeae (Poaceae) based on DNA sequences from chloroplast, mitochondrial, and nuclear genomes. Am. J. Bot. 92, 1548–1558. doi: 10.3732/ajb.92.9.1548 21646172

[B29] Guo Z. T. Ruddiman W. F. Hao Q. Z. Wu H. B. Qiao Y. S. Zhu R. X.. (2002). Onset of Asian desertification by 22 myr ago inferred from loess deposits in China. Nature 416, 159–163. doi: 10.1038/416159a 11894089

[B30] Hall T. A. (1999). BioEdit: a user-friendly biological sequence alignment editor and analysis program for windows 95/98/NT. Nucleic Acids Symposium Ser. 41, 95–98.

[B31] Hall R. (2009). Southeast asia's changing palaeogeography. Blumea 54, 148–161. doi: 10.3767/000651909X475941

[B32] Hantoro W. Faure H. Djuwansah R. Faure-Denard L. Pirazzoli P. J. (1995). The sunda and sahul continental platform: lost land of the last glacial continent in SE Asia. Quaternary Int. 29, 129–134. doi: 10.1016/1040-6182(95)00015-B

[B33] Higgins S. I. Nathan R. Cain M. L. (2003). Are long-distance dispersal events in plants usually caused by nonstandard means of dispersal? Ecology 84, 1945–1956. doi: 10.1890/01-0616

[B34] Huang X. H. Deng T. Moore M. J. Wang H. C. Li Z. M. Lin N.. (2019). Tropical Asian origin, boreotropical migration and long-distance dispersal in nettles (Urticeae, urticaceae). Mol. Phylogenet. Evol. 137, 190–199. doi: 10.1016/j.ympev.2019.05.007 31102687

[B35] Ian M. R. (2006). Northern hemisphere plant disjunctions: a window on tertiary land bridges and climate change? Ann. Bot. 98, 465–472. doi: 10.1093/aob/mcl148 16845136 PMC2803576

[B36] Ji Y. H. Yang L. F. Chase M. W. Liu C. K. Yang Z. Y. Yang J.. (2019). Plastome phylogenomics, biogeography, and clade diversification of *Paris* (Melanthiaceae). BMC Plant Biol. 19, 14. doi: 10.1186/s12870-019-2147-6 31805856 PMC6896732

[B37] Jiang C. Tan K. Ren M. (2017). Effects of monsoon on distribution patterns of tropical plants in Asia. Chin. J. Plant Ecol. 41, 1103–1112. doi: 10.17521/cjpe.2017.0070

[B38] Jin J. J. Yang M. Q. Fritsch P. W. Van Velzen R. Li D. Z. Yi T. S. (2020). Born migrators: historical biogeography of the cosmopolitan family cannabaceae. J. Systematics Evol. 58, 461–473. doi: 10.1111/jse.12552

[B39] Kalyaanamoorthy S. Minh B. Q. Wong T. K. F. Von Haeseler A. Jermiin L. S. (2017). ModelFinder: fast model selection for accurate phylogenetic estimates. Nat. Methods 14, 587–589. doi: 10.1038/nmeth.4285 28481363 PMC5453245

[B40] Kar R. (1996). On the Indian origin of *Ocimum* (Lamiaceae): a palynological approach. Palaeobotanist 43, 43–50.

[B41] Kato M. (2000). Anthophilous insect community and plant-pollinator interactions on amami islands in the Ryukyu archipelago, Japan. Kyoto Univ. 29, 157–254. Available at: http://hdl.handle.net/2433/156116

[B42] Kato M. Shibata A. Yasui T. Nagamasu H. (1999). Impact of introduced honeybees, *Apis mellifera*, upon native bee communities in the bonin (Ogasawara) islands. Res. Population Ecol. 41, 217–228. doi: 10.1007/s101440050025

[B43] Katoh K. Standley D. M. (2013). MAFFT multiple sequence alignment software version 7: improvements in performance and usability. Mol. Biol. Evol. 30, 772–780. doi: 10.1093/molbev/mst010 23329690 PMC3603318

[B44] Kawakubo N. (1990). Dioecism of the genus *Callicarpa* (Verbenaceae) in the bonin (Ogasawara) islands. botanical magazine= Shokubutsu-gaku-zasshi 103, 57–66. doi: 10.1007/BF02488411

[B45] Keigwin L. D. (1978). Pliocene closing of the isthmus of Panama, based on biostratigraphic evidence from nearby pacific ocean and Caribbean Sea cores. Geology 6, 630–634. doi: 10.1130/0091-7613(1978)6<630:PCOTIO>2.0.CO;2

[B46] Kessler-Rios M. M. Kattan G. H. (2012). Fruits of melastomataceae: phenology in Andean forest and role as a food resource for birds. J. Trop. Ecol. 28, 11–21. doi: 10.1017/S0266467411000642

[B47] Kumar S. Stecher G. Tamura K. (2016). MEGA7: molecular evolutionary genetics analysis version 7.0 for bigger datasets. Mol. Biol. Evol. 33, 1870–1874. doi: 10.1093/molbev/msw054 27004904 PMC8210823

[B48] Latham R. E. Ricklefs R. E. (1993). Continental comparisons of temperate-zone tree species diversity. Species Diversity Ecol. communities: historical geographical Perspect II, 294–314.

[B49] Lavin M. Luckow M. (1993). Origins and relationships of tropical north America in the context of the boreotropics hypothesis. Am. J. Bot. 80, 1–14. doi: 10.1002/j.1537-2197.1993.tb13761.x

[B50] Lee T. Y. Lawver L. A. (1995). Cenozoic Plate reconstruction of southeast Asia. Tectonophysics 251, 85–138. doi: 10.1016/0040-1951(95)00023-2

[B51] Leeratiwong C. Chantaranothai P. Paton A. (2007). Notes on the genus *Callicarpa* (Lamiaceae) in Thailand. Thai For. Bull. 37, 73–79. Available at: https://li01.tci-thaijo.org/index.php/ThaiForestBulletin/article/view/24203

[B52] Leeratiwong C. Chantaranothai P. Paton A. J. (2009). A synopsis of the genus *Callicarpa* L.(Lamiaceae) in Thailand. Thai For. Bull. 35, 36–58. Available at: https://li01.tcithaijo.org/index.php/ThaiForestBulletin/article/view/24334

[B53] Le Pechon T. Zhang L. He H. Zhou X. M. Bytebier B. Gao X. F.. (2016). A well-sampled phylogenetic analysis of the polystichoid ferns (Dryopteridaceae) suggests a complex biogeographical history involving both boreotropical migrations and recent transoceanic dispersals. Mol. Phylogenet. Evol. 98, 324–336. doi: 10.1016/j.ympev.2016.02.018 26944012

[B54] Les D. H. Crawford D. J. Kimball R. T. Moody M. L. Landolt E. (2003). Biogeography of discontinuously distributed hydrophytes: a molecular appraisal of intercontinental disjunctions. Int. J. Plant Sci. 164, 917–932. doi: 10.1086/378650

[B55] Li B. Cantino P. D. Olmstead R. G. Bramley G. L. Xiang C. L. Ma Z. H.. (2016). A large-scale chloroplast phylogeny of the lamiaceae sheds new light on its subfamilial classification. Sci. Rep. 6, 34343. doi: 10.1038/srep34343 27748362 PMC5066227

[B56] Li Y. Q. Dressler S. Zhang D. X. Renner S. S. (2009). More Miocene dispersal between Africa and Asia-the case of *Bridelia* (Phyllanthaceae). Systematic Bot. 34, 521–529. doi: 10.1600/036364409789271263

[B57] Li Z. Z. Lehtonen S. Martins K. Gichira A. W. Wu S. Li W.. (2020b). Phylogenomics of the aquatic plant genus *Ottelia* (Hydrocharitaceae): implications for historical biogeography. Mol. Phylogenet. Evol. 152, 106939. doi: 10.1016/j.ympev.2020.106939 32791299

[B58] Li H. W. Liu B. Davis C. C. Yang Y. (2020a). Plastome phylogenomics, systematics, and divergence time estimation of the *Beilschmiedia* group (Lauraceae). Mol. Phylogenet. Evol. 151, 13. doi: 10.1016/j.ympev.2020.106901 32619568

[B59] Li Z. X. Powell C. (2001). An outline of the palaeogeographic evolution of the Australasian region since the beginning of the neoproterozoic. Earth-Science Rev. 53, 237–277. doi: 10.1016/S0012-8252(00)00021-0

[B60] Li P. Qi Z. C. Liu L. X. Ohi-Toma T. Lee J. Hsieh T. H.. (2017). Molecular phylogenetics and biogeography of the mint tribe elsholtzieae (Nepetoideae, lamiaceae), with an emphasis on its diversification in East Asia. Sci. Rep. 7, 12. doi: 10.1038/s41598-017-02157-6 28515478 PMC5435694

[B61] Li R. Wen J. (2013). Phylogeny and biogeography of *Dendropanax* (Araliaceae), an amphi-pacific disjunct genus between tropical/subtropical Asia and the neotropics. Systematic Bot. 38, 536–551. doi: 10.1600/036364413X666606

[B62] Lian L. Ortiz R. D. C. Jabbour F. Zhang C. F. Xiang X. G. Erst A. S.. (2020). Phylogeny and biogeography of pachygoneae (Menispermaceae), with consideration of the boreotropical flora hypothesis and resurrection of the genera *Cebatha* and *Nephroia* . Mol. Phylogenet. Evol. 148, 106825. doi: 10.1016/j.ympev.2020.106825 32294547

[B63] Lindelof K. Lindo J. A. Zhou W. B. Ji X. Xiang Q. Y. (2020). Phylogenomics, biogeography, and evolution of the blue- or white-fruited dogwoods (*Cornus*)-insights into morphological and ecological niche divergence following intercontinental geographic isolation. J. Systematics Evol. 58, 604–645. doi: 10.1111/jse.12676

[B64] Linnaeus C. (1753). Species plantarum. 1st edn (Stockholm, Sweden: Laurentius Salvius).

[B65] Liu J. Q. Wang Y. J. Wang A. L. Hideaki O. Abbott R. J. (2006). Radiation and diversification within the *Ligularia*-*Cremanthodium*-*Parasenecio* complex (Asteraceae) triggered by uplift of the qinghai-Tibetan plateau. Mol. Phylogenet. Evol. 38, 31–49. doi: 10.1016/j.ympev.2005.09.010 16290033

[B66] Liu C. Yang J. Jin L. Wang S. Yang Z. Ji Y. (2021). Plastome phylogenomics of the East Asian endemic genus *Dobinea* . Plant Diversity 43, 35–42. doi: 10.1016/j.pld.2020.05.002 33778223 PMC7987559

[B67] Lohman D. J. De Bruyn M. Page T. Von Rintelen K. Hal R. Ng P. K. L.. (2011). Biogeography of the indo-Australian archipelago. Annu. Rev. Ecology Evolution Systematics 42, 205–226. doi: 10.1146/annurev-ecolsys-102710-145001

[B68] Loiselle B. A. Blake J. G. (1999). Dispersal of melastome seeds by fruit-eating birds of tropical forest understory. Ecology 80, 330–336. doi: 10.1890/0012-9658(1999)080[0330:DOMSBF]2.0.CO;2

[B69] Lomolino M. V. (2010). Four Darwinian themes on the origin, evolution and preservation of island life. J. Biogeography 37, 985–994. doi: 10.1111/j.1365-2699.2009.02247.x

[B70] Ma Z. Bramley G. L. C. Zhang D. (2016). Pollen morphology of *Callicarpa* l. (Lamiaceae) from China and its systematic implications. Plant Systematics Evol. 302, 67–88. doi: 10.1007/s00606-015-1244-8

[B71] Ma W. J. Su Z. W. Ma Z. H. (2022). Chemical constituents of *Callicarpa integerrima* . Guihaia 42, 1–11. doi: 10.11931/guihaia.gxzw202202008

[B72] Ma Z. H. Zhang D. X. (2012). *Callicarpa hainanensis*: a new species of *Callicarpa* from hainan, China. J. Systematics Evol. 50, 573–573. doi: 10.1111/j.1759-6831.2012.00229_1.x

[B73] Manos P. S. Donoghue M. J. (2001). Progress in northern hemisphere phytogeography: an introduction. Int. J. Plant Sci. 162, S1–S2. doi: 10.1086/324421

[B74] Martinez-Millan M. (2010). Fossil record and age of the asteridae. Botanical Rev. 76, 83–135. doi: 10.1007/s12229-010-9040-1

[B75] Miller M. Pfeiffer W. Schwartz T. (2010). Creating the CIPRES science gateway for inference of large phylogenetic trees in proceedings of the gateway. New Orleans: Computing Environments Workshop (GCE) 1–8. doi: 10.1109/GCE.2010.5676129

[B76] Milne R. I. Abbott R. J. (2002). The origin and evolution of tertiary relict floras. Adv. Botanical Res. 38, 281–314. doi: 10.1016/S0065-2296(02)38033-9

[B77] Momose K. Yumoto T. Nagamitsu T. Kato M. Nagamasu H. Sakai S.. (1998). Pollination biology in a lowland dipterocarp forest in Sarawak, Malaysia. I. Characteristics of the plant-pollinator community in a lowland dipterocarp forest. Am. J. Bot. 85, 1477–1501. doi: 10.2307/2446404 21684899

[B78] Myers N. Mittermeier R. A. Mittermeier C. G. Da Fonseca G. Kent J. (2000). Biodiversity hotspots for conservation priorities. Nature 403, 853–858. doi: 10.1038/35002501 10706275

[B79] Nguyen L. T. Schmidt H. A. von Haeseler A. Minh B. Q. (2015). IQ-TREE: a fast and effective stochastic algorithm for estimating maximum-likelihood phylogenies. Mol. Biol. Evol. 32, 268–274. doi: 10.1093/molbev/msu300 25371430 PMC4271533

[B80] Nylander J. (2004). MrModeltest v.2. (Sweden: Evolutionary Biology Centre, Uppsala University).

[B81] Paradis E. Claude J. Strimmer K. (2004). APE: analyses of phylogenetics and evolution in r language. Bioinformatics 20, 289–290. doi: 10.1093/bioinformatics/btg412 14734327

[B82] Popp M. Mirre V. Brochmann C. (2011). A single mid-pleistocene long-distance dispersal by a bird can explain the extreme bipolar disjunction in crowberries (*Empetrum*). Proc. Natl. Acad. Sci. United States America 108, 6520–6525. doi: 10.1073/pnas.1012249108 PMC3081031 21402939

[B83] Qian H. Ricklefs R. E. (2000). Large-Scale processes and the Asian bias in species diversity of temperate plants. Nature 407, 180–182. doi: 10.1038/35025052 11001054

[B84] Qiu Y. X. Fu C. X. Comes H. P. (2011). Plant molecular phylogeography in China and adjacent regions: tracing the genetic imprints of quaternary climate and environmental change in the world's most diverse temperate flora. Mol. Phylogenet. Evol. 59, 225–244. doi: 10.1016/j.ympev.2011.01.012 21292014

[B85] Rambaut A. Drummond A. J. Xie D. Baele G. Suchard M. A. (2018). Posterior summarization in bayesian phylogenetics using tracer 1.7. Systematic Biol. 67, 901. doi: 10.1093/sysbio/syy032 PMC6101584 29718447

[B86] Ronquist F. Teslenko M. van der Mark P. Ayres D. L. Darling A. Hohna S.. (2012). MrBayes 3.2: efficient bayesian phylogenetic inference and model choice across a large model space. Systematic Biol. 61, 539–542. doi: 10.1093/sysbio/sys029 PMC3329765 22357727

[B87] Royden L. H. Burchfiel B. C. van der Hilst R. D. (2008). The geological evolution of the Tibetan plateau. Science 321, 1054–1058. doi: 10.1126/science.1155371 18719275

[B88] Sang T. Crawford D. J. Stuessy T. F. (1997). Chloroplast DNA phylogeny, reticulate evolution, and biogeography of *Paeonia* (Paeoniaceae). Am. J. Bot. 84, 1120–1136. doi: 10.2307/2446155 21708667

[B89] Santosh M. Senshu H. (2011). History of supercontinents and its relation to the origin of Japanese islands. J. geography-chigaku zasshi 120, 100–114. doi: 10.5026/jgeography.120.100

[B90] Shaffer S. A. Tremblay Y. Weimerskirch H. Scott D. Thompson D. R. Sagar P. M.. (2006). Migratory shearwaters integrate oceanic resources across the pacific ocean in an endless summer. Proc. Natl. Acad. Sci. United States America 103, 12799–12802. doi: 10.1073/pnas.060371510 PMC1568927 16908846

[B91] Shaw J. Lickey E. B. Beck J. T. Farmer S. B. Liu W. S. Miller J.. (2005). The tortoise and the hare II: relative utility of 21 noncoding chloroplast DNA sequences for phylogenetic analysis. Am. J. Bot. 92, 142–166. doi: 10.3732/ajb.92.1.142 21652394

[B92] Shaw J. Lickey E. B. Schilling E. E. Small R. L. (2007). Comparison of whole chloroplast genome sequences to choose noncoding regions for phylogenetic studies in angiosperms: the tortoise and the hare III. Am. J. Bot. 94, 275–288. doi: 10.3732/ajb.94.3.275 21636401

[B93] Small R. L. Cronn R. C. Wendel J. F. (2004). Use of nuclear genes for phylogeny reconstruction in plants. Aust. Systematic Bot. 17, 145–170. doi: 10.1071/SB03015

[B94] Smith A. B. Peterson K. J. (2002). Dating the time of origin of major clades: molecular clocks and the fossil record. Annu. Rev. Earth Planetary Sci. 30, 65–88. doi: 10.1146/annurev.earth.30.091201.140057

[B95] Sun Y. Skinner D. Z. Liang G. H. Hulbert S. H. (1994). Phylogenetic analysis of *Sorghum* and related taxa using internal transcribed spacers of nuclear ribosomal DNA. Theor. Appl. Genet. 89, 26–32. doi: 10.1007/BF00226978 24177765

[B96] Sun X. Wang P. (2005). How old is the Asian monsoon system? —palaeobotanical records from China. Palaeogeography Palaeoclimatology Palaeoecol. 222, 181–222. doi: 10.1016/j.palaeo.2005.03.005

[B97] Tan K. Malabrigo Pastor L. Ren M. (2020). Origin and evolution of biodiversity hotspots in southeast Asia. Acta Ecologica Sin. 40, 3866–3877. doi: 10.5846/stxb201904160762

[B98] Tate J. A. Simpson B. B. (2003). Paraphyly of *Tarasa* (Malvaceae) and diverse origins of the polyploid species. Systematic Bot. 28, 723–737. doi: 10.1043/02-64.1

[B99] Thomas D. C. Hughes M. Phutthai T. Ardi W. H. Rajbhandary S. Rubite R.. (2012). West To east dispersal and subsequent rapid diversification of the mega-diverse genus *Begonia* (Begoniaceae) in the malesian archipelago. J. Biogeography 39, 98–113. doi: 10.1111/j.1365-2699.2011.02596.x

[B100] Tiffney B. (1985). The Eocene north Atlantic land bridge: its importance in tertiary and modern phytogeography of the northern hemisphere. J. Arnold Arbor. 66, 243–273. doi: 10.5962/bhl.part.13183

[B101] Tiffney B. H. Manchester S. R. (2001). The use of geological and paleontological evidence in evaluating plant phylogeographic hypotheses in the northern hemisphere tertiary. Int. J. Plant Sci. 162, S3–S17. doi: 10.1086/323880

[B102] Toussaint E. F. A. Hendrich L. Hajek J. Michat M. C. Panjaitan R. Short A. E. Z.. (2017). Evolution of pacific rim diving beetles sheds light on amphi-pacific biogeography. Ecography 40, 500–510. doi: 10.1111/ecog.02195

[B103] Tsukaya H. Fukuda T. Yokoyama J. (2003). Hybridization and introgression between *Callicarpa japonica* and *C. mollis* (Verbenaceae) in central Japan, as inferred from nuclear and chloroplast DNA sequences. Mol. Ecol. 12, 3003–3011. doi: 10.1046/j.1365-294X.2003.01961.x 14629381

[B104] Tu Y. H. Sun L. N. Guo M. L. Chen W. S. (2013). The medicinal uses of *Callicarpa* l. @ in traditional Chinese medicine: an ethnopharmacological, phytochemical and pharmacological review. J. Ethnopharmacology 146, 465–481. doi: 10.1016/j.jep.2012.12.051 23313870

[B105] Van Damme K. Sinev A. Y. (2013). Tropical amphi-pacific disjunctions in the cladocera (Crustacea: branchiopoda). J. Limnology 72, 209–244. doi: 10.4081/jlimnol.2013.s2.e11

[B106] Van Der Pijl L. (1982). “Principles of dispersal in higher plants,” in Springer, vol. 214. (Berlin: Verlag).

[B107] Van Welzen P. C. Parnell J. Slik J. W. F. (2011). Wallace's line and plant distributions: two or three phytogeographical areas and where to group Java? Biol. J. Linn. Soc. 103, 531–545. doi: 10.1111/j.1095-8312.2011.01647.x

[B108] Walker J. Geissman J. Bowring S. Babcock L. (2018). Geologic time scale v. 5.0. Geological Soc. America. Available at: https://www.geosociety.org/.

[B109] Wang Z. (2017). Geological features, the formation and the evolution of the qinghai-Tibetan plateau. Sci. Technol. Rev. 35, 51–58. doi: 10.3981/j.issn.1000-7857.2017.06.005

[B110] Wang W. Xiang X. G. Xiang K. L. Ortiz R. D. C. Jabbour F. Chen Z. D. (2020). A dated phylogeny of lardizabalaceae reveals an unusual long-distance dispersal across the pacific ocean and the rapid rise of East Asian subtropical evergreen broadleaved forests in the late Miocene. Cladistics 36, 447–457. doi: 10.1111/cla.12414 34618951

[B111] Webster P. J. Magana V. O. Palmer T. N. Shukla J. Tomas R. A. Yanai M.. (1998). Monsoons: processes, predictability, and the prospects for prediction. J. Geophysical Research-Oceans 103, 14451–14510. doi: 10.1029/97JC02719

[B112] Wei R. Xiang Q. P. Schneider H. Sundue M. A. Kessler M. Kamau P. W.. (2015). Eurasian Origin, boreotropical migration and transoceanic dispersal in the pantropical fern genus *Diplazium* (Athyriaceae). J. Biogeography 42, 1809–1819. doi: 10.1111/jbi.12551

[B113] Wen J. (1999). Evolution of eastern Asian and eastern north American disjunct distributions in flowering plants. Annu. Rev. Ecol. Systematics 30, 421–455. doi: 10.1146/annurev.ecolsys.30.1.421

[B114] Wen J. (2001). Evolution of eastern Asian-Eastern north American biogeographic disjunctions: a few additional issues. Int. J. Plant Sci. 162, S117–S122. doi: 10.1086/322940

[B115] Wen J. Nie Z. L. Ickert-Bond S. M. (2016). Intercontinental disjunctions between eastern Asia and western north America in vascular plants highlight the biogeographic importance of the Bering land bridge from late Cretaceous to neogene. J. Systematics Evol. 54, 469–490. doi: 10.1111/jse.12222

[B116] White T. J. Bruns T. Lee S. Talyor J. (1990). Amplification and direct sequencing of fungal ribosomal RNA genes for phylogenetics. PCR protocols: guide to Methods Appl. 18, 315–322. doi: 10.1016/B978-0-12-372180-8.50042-1

[B117] Wolfe J. (1975). Some aspects of plant geography of the northern hemisphere during the late Cretaceous and tertiary. Ann. Missouri Botanical Garden 62, 264–279. doi: 10.2307/2395198

[B118] Wu Z. Chen J. Xu F. Wang H. (2018). Research progress on *Callicarpa* plants. J. Guangdong Pharm. Univ. 34, 808–813. doi: 10.16809/j.cnki.2096-3653.2018102101

[B119] Wu X. K. Liu X. Y. Kodrul T. Quan C. Jin J. H. (2019). *Dacrycarpus* pattern shedding new light on the early floristic exchange between Asia and Australia. Natl. Sci. Rev. 6, 1086–1090. doi: 10.1093/nsr/nwz060 34691981 PMC8291558

[B120] Wu S. Wu Z. (1996). A proposal for a new floristic kingdom (realm): the Asiatic kingdom its delineationand characteristics. Floristic Characteristics, 3–42.

[B121] Xu L. Chen F. Xie Y. (2013). Cross-breeding between two species of *Callicarpa* . J. Huazhong Agric. Univ. 32, 23–27. doi: 10.3969/j.issn.1000-2421.2013.04.005

[B122] Yang Y. Li Z. Y. Shao J. J. Wang G. Wen R. Tian J. Z.. (2021). *Callicarpa nudiflora* hook. & am.: a comprehensive review of its phytochemistry and pharmacology. J. Ethnopharmacology 264, 113123. doi: 10.1016/j.jep.2020.113123 32783986

[B123] Yang M. Q. Li D. Z. Wen J. Yi T. S. (2017). Phylogeny and biogeography of the amphi-pacific genus *Aphananthe* . PloS One 12, e0171405. doi: 10.1371/journal.pone.0171405 28170425 PMC5295712

[B124] Yang T. Lu L. M. Wang W. Li J. H. Manchester S. R. Wen J.. (2018). Boreotropical range expansion and long-distance dispersal explain two amphi-pacific tropical disjunctions in sabiaceae. Mol. Phylogenet. Evol. 124, 181–191. doi: 10.1016/j.ympev.2018.03.005 29548980

[B125] Yao G. Drew B. T. Yi T. S. Yan H. F. Yuan Y. M. Ge X. J. (2016). Phylogenetic relationships, character evolution and biogeographic diversification of *Pogostemon* s.l. (Lamiaceae). Mol. Phylogenet. Evol. 98, 184–200. doi: 10.1016/j.ympev.2016.01.020 26923493

[B126] Yu Y. Blair C. He X. (2020). RASP 4: ancestral state reconstruction tool for multiple genes and characters. Mol. Biol. Evol. 37, 604–606. doi: 10.1093/molbev/msz257 31670774

[B127] Yu Y. Harris A. J. Blair C. He X. J. (2015). RASP (Reconstruct ancestral state in phylogenies): a tool for historical biogeography. Mol. Phylogenet. Evol. 87, 46–49. doi: 10.1016/j.ympev.2015.03.008 25819445

[B128] Yu X. Q. Maki M. Drew B. T. Paton A. J. Li H. W. Zhao J. L.. (2014). Phylogeny and historical biogeography of *Isodon* (Lamiaceae): rapid radiation in south-west China and Miocene overland dispersal into Africa. Mol. Phylogenet. Evol. 77, 183–194. doi: 10.1016/j.ympev.2014.04.017 24792085

[B129] Yuan Y. M. Wohlhauser S. Möller M. Klackenberg J. Callmander M. W. Küpfer P. (2005). Phylogeny and biogeography of *Exacum* (Gentianaceae): a disjunctive distribution in the Indian ocean basin resulted from long distance dispersal and extensive radiation. Syst. Biol. 54, 21–34. doi: 10.1080/10635150590905867 15805008

[B130] Zachos J. Pagani M. Sloan L. Thomas E. Billups K. (2001). Trends, rhythms, and aberrations in global climate 65 ma to present. Science 292, 686–693. doi: 10.1126/science.1059412 11326091

[B131] Zhang D. Gao F. L. Jakovlic I. Zou H. Zhang J. Li W. X.. (2020a). PhyloSuite: an integrated and scalable desktop platform for streamlined molecular sequence data management and evolutionary phylogenetics studies. Mol. Ecol. Resour. 20, 348–355. doi: 10.1111/1755-0998.13096 31599058

[B132] Zhang M. H. Wang C. Y. Zhang C. Zhang D. G. Li K. G. Nie Z. L.. (2020b). Phylogenetic relationships and biogeographic history of the unique *Saxifraga* sect. *Irregulares* (Saxifragaceae) from eastern Asia. J. Systematics Evol. 58, 958–971. doi: 10.1111/jse.12547

[B133] Zhou L. L. Su Y. C. F. Thomas D. C. Saunders R. M. K. (2012). Out-of-Africa' dispersal of tropical floras during the Miocene climatic optimum: evidence from *Uvaria* (Annonaceae). J. Biogeography 39, 322–335. doi: 10.1111/j.1365-2699.2011.02598.x

[B134] Zhou W. B. Xiang Q. Y. Wen J. (2020). Phylogenomics, biogeography, and evolution of morphology and ecological niche of the eastern Asian-eastern north American *Nyssa* (Nyssaceae). J. Systematics Evol. 58, 571–603. doi: 10.1111/jse.12599

