# Supplementary material for: Phylogenetic relationships and biogeography of Asia Callicarpa (Lamiaceae), with consideration of a long-distance dispersal across the Pacific Ocean —insights into divergence modes of pantropical flora
Source: Front Plant Sci. 2023 May 15;14:1133157. doi: 10.3389/fpls.2023.1133157 (PMC10225572; doi:10.3389/fpls.2023.1133157)
Supplement: Supplementary file 1 [file DataSheet_1.docx]

Supplementary Material

Phylogenetic relationships and biogeography of Asia *Callicarpa* (Lamiaceae), with consideration of a long-distance dispersal across the Pacific Ocean —insights into divergence modes of Pantropical flora

Huimin Cai, Xing Liu, Wenqiao Wang, Zhonghui Ma*, Bo Li, Gemma L. C. Bramley, Dianxiang Zhang

*** Correspondence:** Corresponding Author: [mazhonghui@gxu.edu.cn](mailto:mazhonghui@gxu.edu.cn)

# Supplementary Figures and Tables

## Supplementary Figures


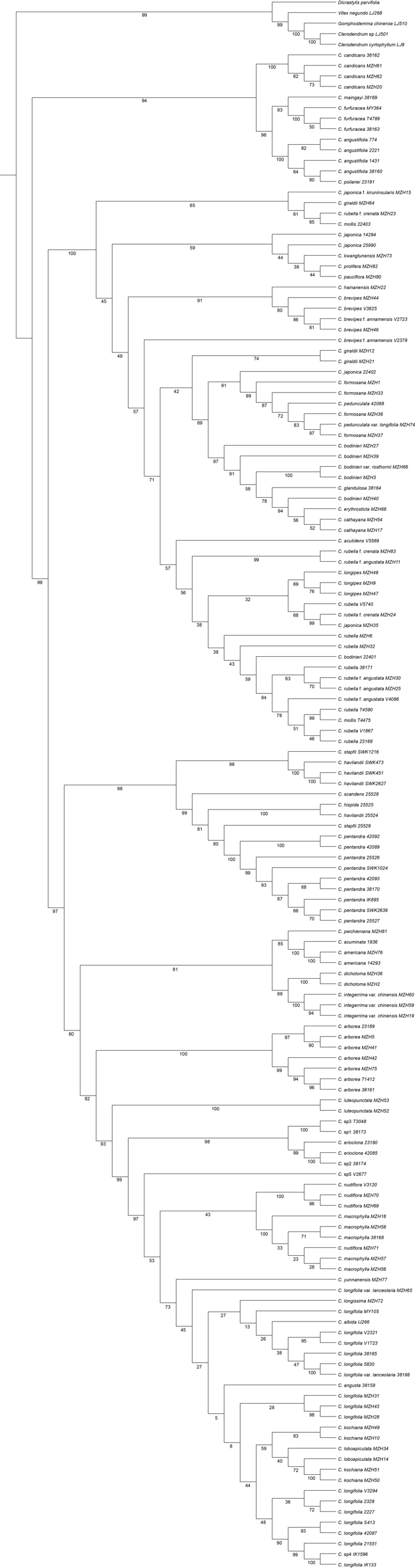

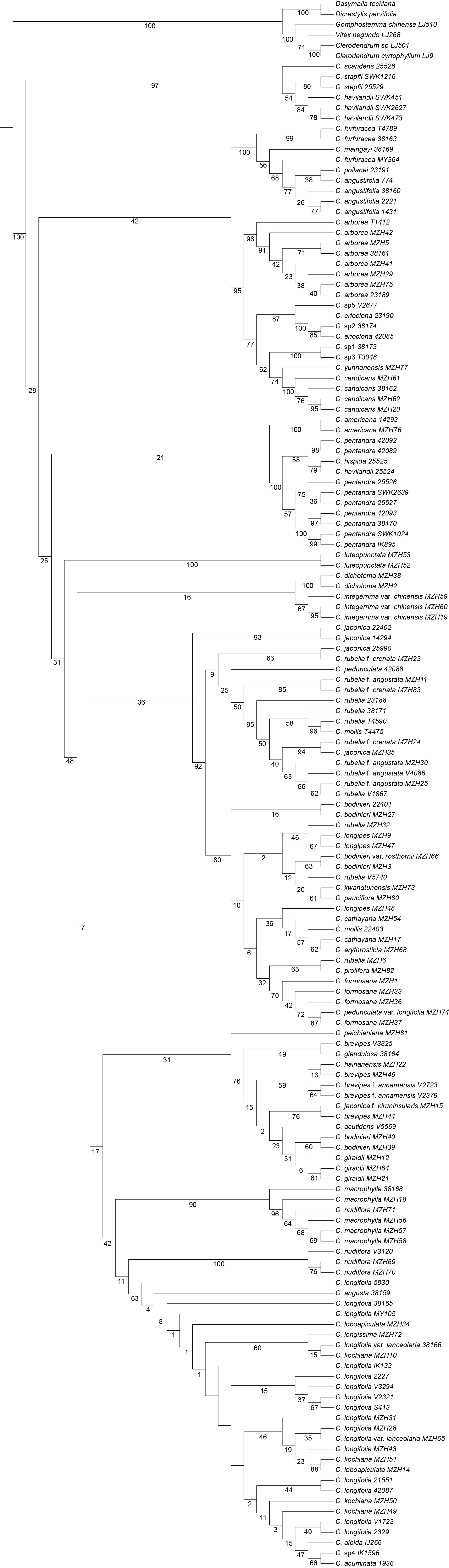


**Supplementary Figure 1.** 1. Maximum likelihood tree derived from cpDNA (eight chloroplast regions); 2. Maximum likelihood tree derived from nrDNA (two nuclear regions).

**
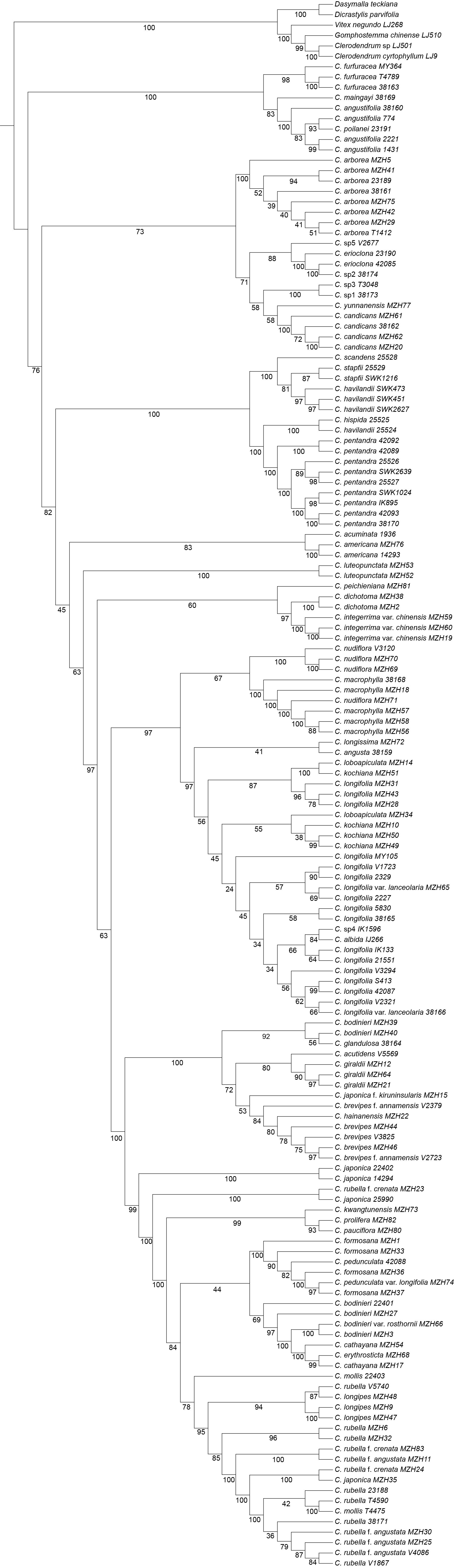

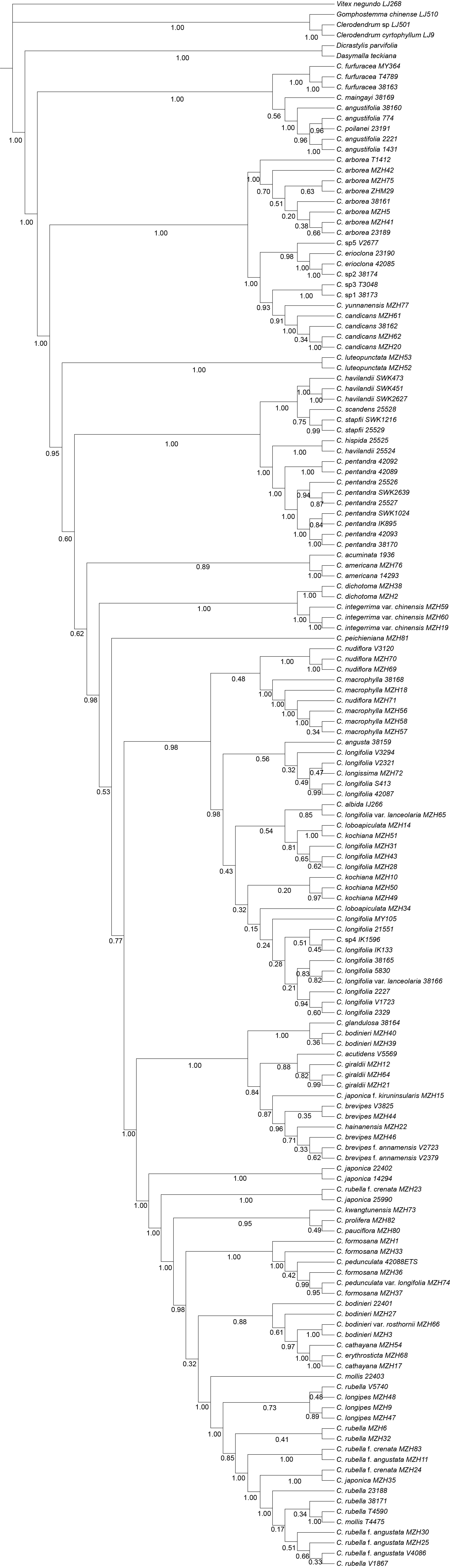
**

**Supplementary Figure 2.** 1. The phylogenetic trees using bayesian inference analyses based combined nrDNA and cpDNA (two nuclear and eight chloroplast regions); 2. The phylogenetic trees using maximum likelihood analyses based combined nrDNA and cpDNA (two nuclear and eight chloroplast regions).

## Supplementary Tables

| **Table S1 Primers used to amplify and sequence nrDNA and cpDNA in this study.** | | | |
| --- | --- | --- | --- |
| **Marker** | **Primer** | **Sequence (5’ to 3’)** | **Reference** |
| ITS | 17SE | ACGAATTCATGGTCCGGTGAAGTGTTCG | Sun et al., 1994 |
|  | 26SE | TAGAATTCCCCGGTTCGCTCGCCGTTAC |  |
|  | ITS-4 | TCCTCCGCTTATTGATATGC | White et al., 1990 |
|  | ITS-5 | GGAAGTAAAAGTCGTAACAAGG |  |
| ETS | 18S-IGS | GAGACAAGCATATGACTACTGGCAGGATCAACCAG | Baldwin and Markos, 1998 |
|  | ETS-B | ATAGAGCGCGTGAGTGGTG | Beardsley and Olmstead., 2002 |
| *matK* | 323f | ATTNTCAAATCNTAKCAGAGGGG | Andersson, 2006 |
|  | 1189r | CGGCTTACTAATRGGATGCCC |  |
| *psbJ*-*petA* | psbJ | ATAGGTACTGTARCYGGTATT | Shaw et al., 2007 |
|  | petA | AACARTTYGARAAGGTTCAATT |  |
|  | psbJF1 | TAGGTTCATCCCTGTAGT | Designed here |
|  | petAR1 | TTTTGTTGTCAATTATGT |  |
| *rpl32*-*trnL* | trnL(UAG) | CTGCTTCCTAAGAGCAGCGT | Shaw et al., 2007 |
|  | rpL32-F | CAGTTCCAAAAAAACGTACTTC |  |
| *trnD*-*trnT* | trnDF | ACCAATTGAACTACAATCCC | Demesure et al., 1995 |
|  | trnT | CTACCACTGAGTTAAAAGGG |  |
| *trnG*-*trnS* | trnS (GCU) | AGATAGGGATTCGAACCCTCGGT | Shaw et al., 2005 |
|  | 5’trnG2S | TTTTACCACTAAACTATACCCGC |  |
| *trnH*-*psbA* | psbA F | GTTATGCATGAACGTAATGCTC | Sang et al., 1997 |
|  | trnH2 | CGCGCATGGTGGATTCACAATCC | Tate & Simpson 2003 |
| *trnQ*-5'*rps16* | trnQ(UUG) | GCGTGGCCAAGYGGTAAGGC | Shaw et al., 2007 |
|  | rps16x1 | GTTGCTTTYTACCACATCGTTT |  |
| 3'*trnV*-*ndhC* | trnV(UAC) x2 | GTCTACGGTTCGARTCCGTA | Shaw et al., 2007 |
|  | ndhC | TATTATTAGAAATGYCCARAAAATATCATATTC |  |
|  | ndhC-f | AGACCATTCCAATGCCCCCTTTCGCC | Dong et al., 2012 |
|  | trnV-r | GTTCGAGTCCGTATAGCCCTA |  |

| **Table S2 Best fit partition model for Iqtree/Mrbayes analysis.** | | |
| --- | --- | --- |
|  | **Partition** | **Model** |
| Iqtree | ETS | GTR+F+R3 |
|  | ITS | GTR+F+R3 |
|  | *psbJ-petA* | K3Pu+F+R2 |
|  | *rpl32-trnL* | TVM+F+R3 |
|  | *trnG-trnS* | TVM+F+R3 |
|  | *trnQ-5’rps16* | TVM+F+R3 |
|  | *trnD-trnT* | K3Pu+F+R2 |
|  | *matK* | K3Pu+F+R2 |
|  | *trnH-psbA* | TN+F+R3 |
|  | *3’trnV-ndhC* | TPM2u+F+G4 |
|  |  |  |
| Mrbayes | ETS | GTR+F+I+G4 |
|  | ITS | GTR+F+I+G4 |
|  | *psbJ-petA* | GTR+F+G4 |
|  | *rpl32-trnL* | GTR+F+G4 |
|  | *trnG-trnS* | GTR+F+G4 |
|  | *trnD-trnT* | HKY+F+G4 |
|  | *matK* | HKY+F+G4 |
|  | *trnH-psbA* | F81+F+G4 |
|  | *trnQ-5’rps16* | GTR+F+G4 |
|  | *3’trnV-ndhC* | GTR+F+G4 |

| **Table S3 Substitution model for each partition.** | |
| --- | --- |
| **Partition** | **Substitution model** |
| ITS | GTR+G |
| ETS | GTR+I+G |
| *matK* | GTR+G |
| *psbJ*-*petA* | GTR+G |
| *rpl32*-*trnL* | GTR+G |
| *trnD*-*trnT* | HKY+G |
| *trnG*-*trnS* | GTR+G |
| *trnH*-*psbA* | HKY+G |
| *trnQ*-5'*rps16* | GTR+G |
| 3'*trnV*-*ndhC* | GTR+G |

| **Table S4 Ages of main lineages and species of *Callicarpa* based on our BEAST analysis.** | | | | |
| --- | --- | --- | --- | --- |
| **Major species** | **Ages (Ma)** | **95%HPD (Ma)** | **Crown age (Ma)** | **95% HPD of crown age (Ma)** |
| *C. maingayi* | 9.92 | 1.34, 22.17 | 16.51 | 3.42, 33.78 |
| *C. furfuracea* | 9.92 | 1.34, 22.17 |  |  |
| *C. poilanei* | 4.37 | 0.04, 12.99 |  |  |
| *C. angustifolia* | 4.37 | 0.04, 12.99 |  |  |
|  |  |  |  |  |
| *C. americana* | 27.9 | 12.89, 41.67 | 27.9 | 12.89, 41.67 |
| *C. acuminata* | 27.9 | 12.89, 41.67 |  |  |
|  |  |  |  |  |
| *C. arborea* | 17.03 | 7.35, 28.3 | 17.03 | 7.35, 28.3 |
| *C.* sp5 | 9.11 | 2.68, 17.55 |  |  |
| *C.* sp2 | 2.72 | 0.26, 6.92 |  |  |
| *C. erioclona* | 2.72 | 0.26, 6.92 |  |  |
| *C.* sp1 | 4.02 | 0.56, 9.26 |  |  |
| *C.* sp3 | 4.02 | 0.56, 9.26 |  |  |
| *C. yunnanensis* | 8.73 | 1.89, 15.42 |  |  |
| *C. candicans* | 8.73 | 1.89, 15.42 |  |  |
|  |  |  |  |  |
| *C. stapfii* | 15.31 | 3.02, 19.56 | 16.14 | 6.88, 26.98 |
| *C. scandens* | 15.31 | 3.02, 19.56 |  |  |
| *C. pentandra* | 7.01 | 1.68, 14.33 |  |  |
| *C. havilandii* | 2.1 | 0.05, 5.64 |  |  |
| *C. hispida* | 2.1 | 0.05, 5.64 |  |  |
|  |  |  |  |  |
| *C. luteopunctata* | 23.75 | 13.12, 36.09 |  |  |
| *C. peichieniana* | 13.4 | 2.56, 23.47 |  |  |
| *C. integerrima* var. *chinensis* | 8.19 | 1.17, 18.16 |  |  |
| *C. dichotoma* | 8.19 | 1.17, 18.16 |  |  |
|  |  |  |  |  |
| *C. macrophylla* | 8.01 | 0.68, 17.71 | 16.51 | 9.48, 25.16 |
| *C. nudiflora* | 8.01 | 0.68, 17.71 |  |  |
| *C. longissima* | 8.73 | 0.58, 15.88 |  |  |
| *C. angusta* | 8.73 | 0.58, 15.88 |  |  |
| *C.* sp4 | 9.57 | 5.24, 14.86 |  |  |
| *C. albida* | 5.78 | 1.72, 10.2 |  |  |
| *C. longifolia* var. *lanceolaria* | 5.78 | 1.72, 10.2 |  |  |
| *C. longifolia* | 8.56 | 0.06, 13.09 |  |  |
| *C. loboapiculata* | 4.97 | 0.02, 11.3 |  |  |
| *C. kochiana* | 4.97 | 0.02, 11.3 |  |  |
|  |  |  |  |  |
| *C. japonica* f. *kiruninsularis* | 10.04 | 1.45, 16.34 | 13.53 | 6.46, 22.37 |
| *C. brevipes* f*. annamensis* | 5.57 | 0.83, 12.26 |  |  |
| *C. hainanensis* | 3 | 0.1, 7.35 |  |  |
| *C. brevipes* | 3 | 0.1, 7.35 |  |  |
| *C. acutidens* | 7.96 | 1.51, 13.37 |  |  |
| *C. giraldii* | 4.8 | 0.67, 10.69 |  |  |
| *C. bodinieri* | 1.91 | 0.03, 5.34 |  |  |
| *C. glandulosa* | 1.91 | 0.03, 5.34 |  |  |
| *C. rubella* f. *crenata* | 8.71 | 3.57, 15.39 |  |  |
| *C. japonica* | 3.9 | 0.65, 8.46 |  |  |
| *C. rubella* f. *angustata* | 1.41 | 0.07, 3.75 |  |  |
| *C. rubella* | 1.41 | 0.07, 3.75 |  |  |
| *C. longipes* | 5.26 | 0.27, 7,11 |  |  |
| *C. mollis* | 5.26 | 0.27, 7,11 |  |  |
| *C. prolifera* | 2.42 | 0.2, 5.07 |  |  |
| *C. pauciflora* | 1.13 | 0, 2.32 |  |  |
| *C. kwangtunensis* | 1.13 | 0, 2.32 |  |  |
| *C. bodinieri* var. *rosthornii* | 2.91 | 0.38, 4.86 |  |  |
| *C. erythrosticta* | 0.86 | 0.03, 2.35 |  |  |
| *C. cathayana* | 0.86 | 0.03, 2.35 |  |  |
| *C. pedunculata* | 2.64 | 0.66, 5.28 |  |  |
| *C. pedunculata* var. *longifolia* | 1.5 | 0.16, 3.33 |  |  |
| *C. formosana* | 1.5 | 0.16, 3.33 |  |  |
